# Long-term lymphoid progenitors independently sustain naïve T and NK cell production in humans

**DOI:** 10.1038/s41467-021-21834-9

**Published:** 2021-03-12

**Authors:** Natalia Izotova, Christine Rivat, Cristina Baricordi, Elena Blanco, Danilo Pellin, Eleanor Watt, Athina S. Gkazi, Stuart Adams, Kimberly Gilmour, Jinhua Bayford, Claire Booth, H. Bobby Gaspar, Adrian J. Thrasher, Luca Biasco

**Affiliations:** 1 Great Ormond Street Institute of Child Health Faculty of Population Health Sciences, London, UK; 2 Gene Therapy Program, Dana-Farber/Boston Children’s Cancer and Blood Disorders Center, Harvard Medical School, Boston, MA USA; 3 Great Ormond Street Hospital, London, UK; 4 Present Address: Orchard Therapeutics, University College of London (UCL), London, UK

**Keywords:** T cells, Haematopoietic stem cells

## Abstract

Our mathematical model of integration site data in clinical gene therapy supported the existence of long-term lymphoid progenitors capable of surviving independently from hematopoietic stem cells. To date, no experimental setting has been available to validate this prediction. We here report evidence of a population of lymphoid progenitors capable of independently maintaining T and NK cell production for 15 years in humans. The gene therapy patients of this study lack vector-positive myeloid/B cells indicating absence of engineered stem cells but retain gene marking in both T and NK. Decades after treatment, we can still detect and analyse transduced naïve T cells whose production is likely maintained by a population of long-term lymphoid progenitors. By tracking insertional clonal markers overtime, we suggest that these progenitors can support both T and NK cell production. Identification of these long-term lymphoid progenitors could be utilised for the development of next generation gene- and cancer-immunotherapies.

## Abstract (web summary)

Gene therapy (GT) using haematopoietic stem cells (HSCs) provides an opportunity to trace cell fates in humans, in vivo. Here the authors present evidence in GT patients for a long term lymphoid progenitor population, surviving and maintaining de novo T and NK cell production for years, independently from HSCs.

## Introduction

Historically, survival and activity of individual human hematopoietic progenitor subtypes have been studied exclusively via transplantation in permissive mouse models^1–3^. Despite being a relevant investigational tool, these models do not recapitulate the human hematopoietic milieu^1^, and production of human T and NK cells is severely compromised. It has therefore been impossible to confidently measure genuine in vivo dynamics of human T/NK lymphoid progenitors^4^. Hematopoietic stem cell (HSC) gene therapy (GT) using integrating viral vectors has opened a unique opportunity to trace the fate of transplanted cells for the first time directly in vivo in humans by means of integration site (IS) clonal tracking^5–7^. From our most recent analysis in patients treated with hematopoietic stem and progenitor cell (HSPC) GT^8^, we predicted mathematically that a population of human lymphoid progenitors might exist which is capable of surviving long-term (Lt) in the absence of ongoing contributions from HSC. Using a similar approach, others have also recently confirmed a diversity of HSPC outputs in different disease settings and aligned with our prediction of a Lt lymphoid-biased cell production, which held true even when complex statistical controls are put in place to obviate restricted sample availability, technical biases and cross-sample contamination effects^9^. However, to date, no experimental setting has been available to validate such a mathematical prediction neither in the mouse nor in humans. We have now studied a unique group of patients in whom Lt lymphoid recovery is sustained in the absence of HSC engraftment. X-linked severe combined immunodeficiency (SCID-X1) is caused by deficiency of the common cytokine receptor gamma chain, a component of multiple cytokine receptors (IL2, IL4, IL7, IL9, IL15 and IL21) responsible for many aspects of lymphoid development and function^10,11^. Infants with SCID-X1 classically present with complete absence of T and NK cells, with normal numbers of intrinsically dysfunctional B cells^12^. Patients with SCID-X1 treated in some of the earliest successful ex vivo GT trials using gammaretroviral vectors now have more than 15 years of follow up (Supplementary Table 1)^13,14^. Importantly, although they received transduced CD34+ HSPCs, no conditioning was administered based on the rationale that corrected T cells would have a very potent growth and survival advantage, and that the longevity of the emergent T cell population would provide major clinical benefit. Of 10 patients treated all are alive with persistence of functional transduced peripheral cells.

Here we show that, in the absence of myelosuppressive conditioning, SCID-X1 patients display no vector-positive myeloid/B cells indicating absence of engineered HSC engraftment but instead retain high level gene marking in both T and NK cells for up to 19 years. Nonetheless, through a comprehensive Lt immunophenotypic, molecular and functional characterisation we show that we still are able to detect bona fide vector-positive naïve T cells, accompanied by maintenance of thymic activity and high T-cell receptor (TCR) diversity in naïve T cells overtime. In the absence of gene corrected HSC, T cell production is therefore likely maintained de novo in these patients by a population of long-term lymphoid progenitors (LtLP). IS analysis further demonstrates in vivo clonal stability/function of LtLP overtime in the absence of insertional mutagenesis. On the basis of NK cell immunophenotyping and molecular tracking we also suggest that these progenitors could be supporting Lt bipotent T/NK-specific output in these patients.

## Results

### Immunophenotypic and molecular follow up of SCID-X1 GT patients

**Fig. 1:**
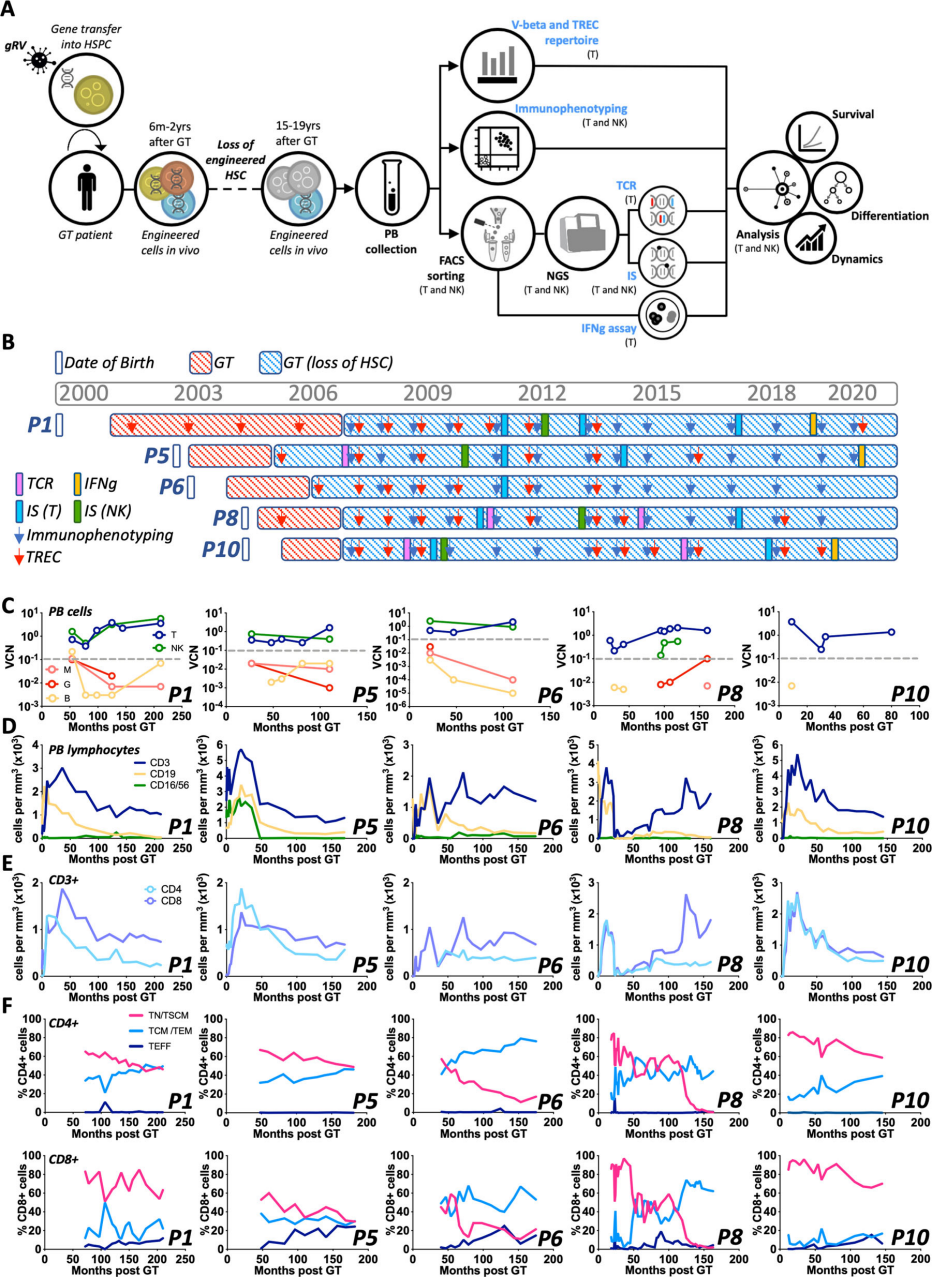
Experimental workflow and molecular/immunophenotypic profile of patients’ blood cells. **A** Schematic of the study design (gRV = gammaRetroviral vector; HSPC hematopoietic stem/progenitor cells, GT gene therapy, HSC hematopoietic stem cells, PB peripheral blood, NGS next generation sequencing, TCR T-cell receptors, IS integration sites). **B** Gantt chart describing patients follow up and approximate dates when assays were conducted. TCR T cell receptor sequencing (pink rectangle), IFNg interferon gamma secretion assay (orange rectangle), IS (T) integration site analysis in T cells (blue rectangle), IS (NK) integration site analysis in NK cells (green rectangle), Immunophenotyping is shown by blue arrows, TREC T cell receptor excision circle content (red arrow). **C** Vector copy number (VCN) measured overtime in T cells (T, dark blue), NK cells (NK, green), B cells (B, yellow), Monocytes (M, salmon) and Neutrophils (N, red) of the five patients object of this study (P1, P5, P6, P8 and P10). The grey dotted line shows background threshold for VCN assay. **D** Absolute counts of T (CD3+, dark blue), B (CD19+, yellow), NK (CD56/16+, green) lymphocytes in peripheral blood overtime. **E** Absolute counts of CD3+CD4+ (light blue) and CD3+CD8+ (blue) cells in peripheral blood overtime. **F** Percentages of naïve T cells (TN)/T memory stem cells (TSCM) precursors (pink), T central memory (TCM)/T effector memory (TEM) (light blue), T effector cells (TEFF) (dark blue) in CD4+ (upper panels) or CD8+ (lower panels) cells overtime.

To assess survival capability, differentiation potential and clonal dynamics of LtLP we designed an analytical workflow combining immunophenotypic, functional and high-throughput molecular assays on samples collected from five SCID-X1 patients, 2.9–18 years after GT (Fig. 1A). As part of the normal follow-up protocol of our SCID-X1 patients, immunophenotyping and vector copy number (VCN) in different blood cell lineages is analysed at a number of time points post treatment (Fig. 1B). Peripheral blood mononuclear cells (PBMCs) from each patient were routinely sorted into five different lineages (neutrophils, T cells, B cells, NK cells and Monocytes) using fluorescence-activated cell sorting (FACS). Quantitative PCR (qPCR) demonstrated that the average copy number for T and NK cells was 2.1 and 2.3 copies per cell, respectively, while in B cells and myeloid cells, VCN remained at the background level since 22–27 months after GT when we started these measurements (Fig. 1C). Because it is known that maintenance of circulating myeloid and naïve B cells is dependent on a continuous output from the bone marrow these data are consistent with a permanent loss or failure of engraftment of engineered long-term (Lt) HSC. Therefore, we initially reasoned that our results could be explained by the survival of vector-marked long-lived circulating peripheral lymphocytes. To test this hypothesis, we investigated the dynamics of the lymphocyte reconstitution over time. We firstly looked at absolute numbers of different immune cell lineages in peripheral blood including T cells (CD3+), B cells (CD19+) and NK cells (CD16/CD56+) (Fig. 1D). Overall, during the early immune reconstitution phase (within the first 6 months), all five patients showed CD3+ cell numbers within normal range, and these numbers remained at or just below the normal range at latest follow up. CD4 and CD8 T cells were present throughout the time of observation although an inverted CD4/CD8 T cell ratio was detected in all patients (Fig. 1E).

To explore the composition of circulating vector positive T cells in these patients, we performed a standard immunophenotypic analysis. Initially, we used CD62L and CD27 cell surface markers to identify a double positive (DP) mixed T cell precursor population composed of naïve T cells (TN) and stem cell memory T cells (TSCM), a central and effector memory T cells (TCM/TEM) population and effector T cells (TEFF) (Fig. 1F). In both CD4 and CD8 subsets, T cell precursors (CD3+ CD62L+ CD27+) were maintained over time up until the latest follow up in a relatively stable fashion in all patients except in patient 8 (P8). In this patient, T cell precursors were preserved up until month 100 post GT, but rapidly declined after this time point, until their relative percentage dropped to <1% of total CD4/CD8 populations. Of note P8 developed T-acute lymphoblastic leukaemia (T-ALL) at 24 months post GT and subsequently underwent a 3-year chemotherapy regimen, achieving long-term clinical and molecular remission^13^. We suspect that this burden of chemotherapy may well be a significant contributor to the observed sharp decline of T cell precursor populations in this particular patient at around 5 years post remission, underlining the biological consistency of our immunophenotypic analyses.

### Immunophenotypic and functional characterisation of bona fide naïve T cells

**Fig. 2:**
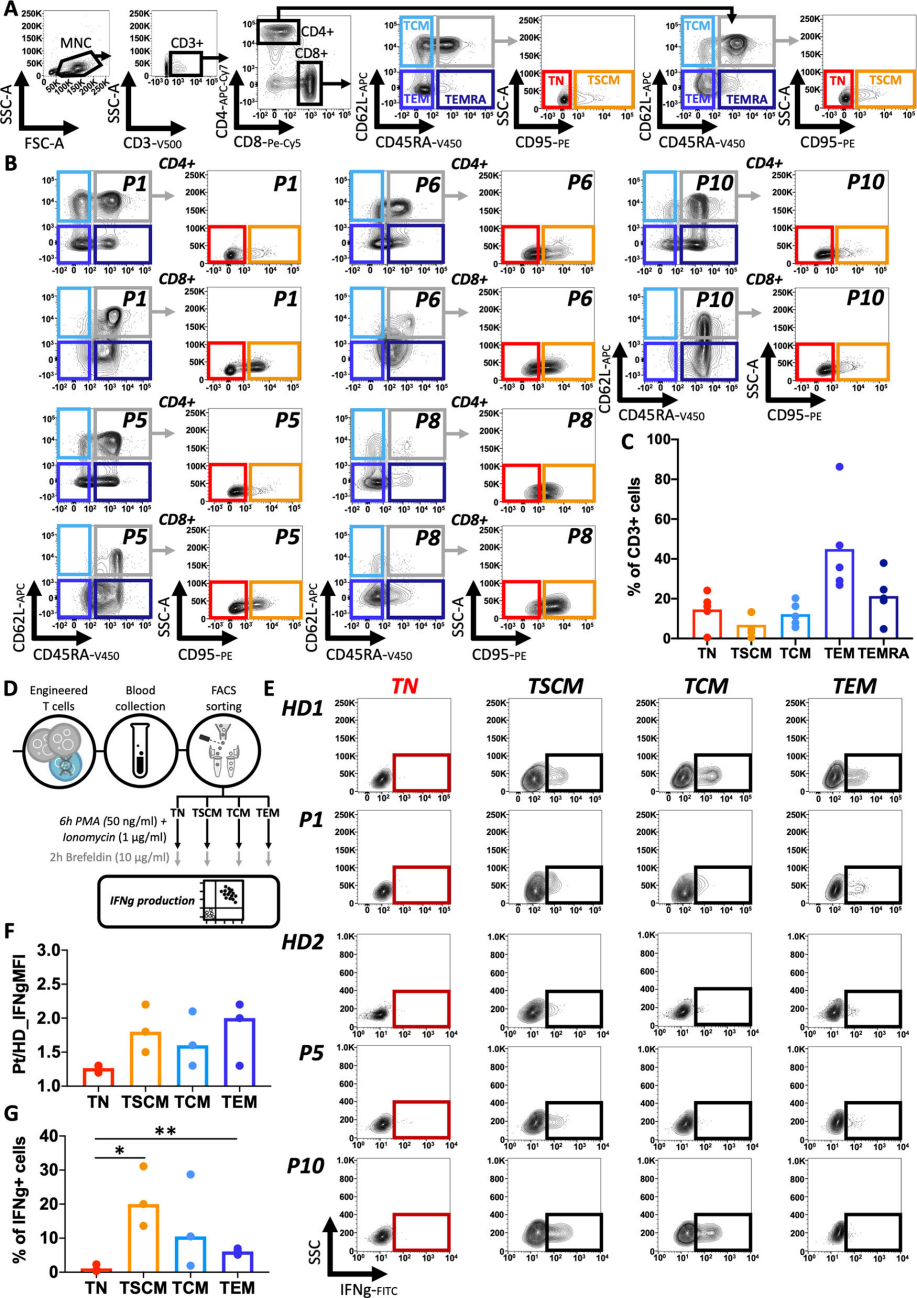
Immunophenotypic and functional validation for the identification of naïve T cells. **A** Gating strategy used for identifying naïve T cells (TN), T memory stem cells (TSCM), T central memory (TCM), T effector memory (TEM) and T effector memory CD45RA+ (TEMRA) subpopulations inside the CD4+ or CD8+ T cells (data displayed are from a healthy donor used as reference). **B** FACS plot showing CD4+ and CD8+ T cell composition at the latest follow up for each patient. **C** Percentage of TN, TSCM, TCM, TEM and TEMRA inside the CD3+ population at latest follow up in the five patients analysed. **D** Experimental scheme for Interferon gamma (IFNg) production assay on four sorted T cell subpopulations. **E** FACS plot showing IFNg expression after PMA/Ionomycin stimulation in each sorted subpopulation from two healthy donors (HD1 and HD2) and three patients (P1, P5 and P10). **F** Ratio between IFNg mean fluorescence intensity (MFI) measured in patients vs. healthy donors T-cell subtypes after stimulation. **G** Percentage of IFNg-positive cells measured in each T-cell subtype from patients after stimulation (unpaired two-tailed *t*-test *p < 0.05 [TN vs. TSCM *p* = 0.0165], **p < 0.01[TN vs. TEM *p* = 0.0033]).

The presence of T cell precursors up to the latest time points was surprising but could be explained by persistence of TSCM which are endowed with decade-long survival in humans^15–18^. To address this point, we used six cell surface markers (CD3, CD4, CD8, CD45RA, CD95 and CD62L) to identify DP precursor (CD3+ CD4/CD8+ CD62L+ CD45RA+), TCM (CD3+ CD4/CD8+ CD62L+ CD45RA−), TEM (CD3+ CD4/CD8+ CD62L− CD45RA−) and TEMRA (CD3+ CD4/CD8+ CD62L− CD45RA+) subsets (Fig. 2A). Most of the T cells exhibited one of the three classical memory phenotypes, TCM, TEM or TEMRA (Fig. 2B). We then used CD95 to differentiate between the two subsets that comprise the DP precursor population of T cells, TN (CD95−) and TSCM (CD95+)^19^. Surprisingly, in 4 out of 5 patients, the majority of cells within the DP population still displayed a TN CD95− phenotype up to the latest follow up (Fig. 2C) in both CD4+ and CD8+ populations (Supplementary Fig. 1). Interestingly, although P8 had a substantially lower numbers of DP cells, 49% of this population was also composed by true TN CD95− (0.5% of total CD3+ cells) (Fig. 2B). Most importantly, we could establish, by means of VCN evaluation on isolated T cell subtypes, that all CD95− TN were vector positive up to the latest follow-up available and in all patients (average VCN of 1.6, Supplementary Fig. 2). To functionally validate the detection of true TN cells we FACS sorted four T cells subsets (TN, TSCM, TCM and TEM) from three patients and from healthy donors (HD), and stimulated them for 6 h with Phorbol 12-myristate 13-acetate (PMA) (50 ng/µl) and ionomycin (1 µg/ml), before adding Brefeldin A (10 µg/ml) and staining for IFN-gamma (IFNg) (Fig. 2E). All patients displayed IFNg expression similar to HD controls (Fig. 2F). TN CD95− vector positive T cells, did not secrete significant levels of IFN-gamma upon stimulation as compared to the other subsets indicating that these were genuine TN cells (Fig. 2G).

### Evaluation of thymic activity and TCR repertoire in GT patients

**Fig. 3:**
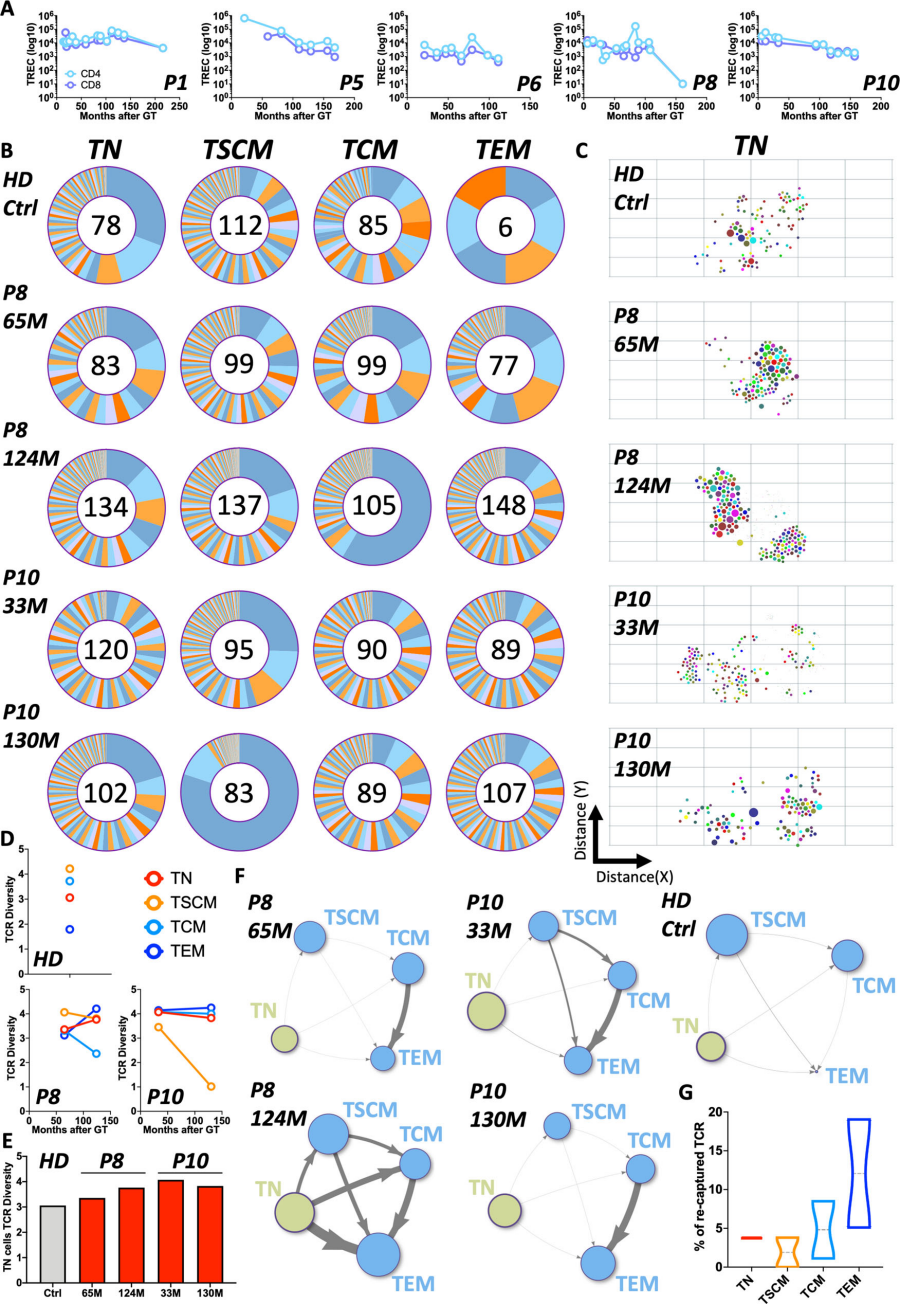
Assessment of thymic activity in GT patients. **A** T-cell receptor excision circles (TREC) content measured in CD4+ and CD8+ cells in five patients overtime. **B** Ring plots displaying the relative fractions of TCR rearrangements detected within each T-cell subtype sorted from a healthy donor control (HD) and two patients (P8 and P10) at two timepoints each (months = M) after GT. Numbers inside each ring correspond to the numbers of individual TCR rearrangements retrieved from each sample. **C** Diversity of TCR rearrangements shown as a 2-dimension t-SNE plot. Coordinates are derived from computing the differences (distance) among clones in sequence composition of a 50 bp window centred on the Complementarity-determining region 3 (CDR3). The size of each dot is proportional to the abundance of the corresponding TCR rearrangement. In these graphical representations the higher it is the spread of dots across the area of the plot the higher it is the diversity of rearrangements observed within the TCR locus. **D** TCR diversity measured by Shannon Diversity index in each sample and T-cell subtypes of HD, P8 and P10. **E** Diversity of TCR repertoire in naïve T cells (TN) of HD (grey bar) and Patients (red bars) at different timepoints). **F** Networks displaying TCR sharing between the four T-cell subtypes from HD or P8 and P10 at each follow up. Nodes represent T-cell subtypes while edges (arrows) represent degree of TCR sharing. Size of each node is proportional to the number of TCR rearrangements detected in each T-cell subtype (Naïve T cells [TN] in light green, other subtypes in light blue). The thickness of the arrows is proportional to the Pearson correlation coefficient calculated on the basis of TCR sharing between each pair of T-cell subtype. **G** Violin plots showing percent of TCR recaptured within each T-cell subtype isolated from two patients at two independent timepoints.

The existence of bona fide vector-positive TN cells suggested that a de novo production of T cells was still occurring many years after the loss of transplanted and transduced HSC. We next wanted to rule out the possibility that we were detecting aberrant long-lived T cells retaining partial TN characteristics and verify instead that we were observing the result of ongoing T cell output. Because new naïve T cells can only be produced in the thymus, we first looked at T-cell receptor excision circle (TREC) content as a surrogate measure of thymic activity (Fig. 3A). Consistent with the presence of genuine TN cells, we observed detectable TREC levels in 4 out of 5 patients up to the latest timepoints suggesting that an active thymic output was maintained years after loss of gene-corrected HSC. P8 showed, as expected, a significant drop in TREC content at the latest follow up, consistent with his decreasing numbers of DP precursors and TN cells. A second marker of physiological thymic activity is a normal distribution of TCR repertoire. We assessed the TCR repertoire composition of the whole T cell population in these patients by Vbeta spectratyping. This was normal in all patients, again except for P8 whose profile was more oligoclonal (Supplementary Fig. 3). These results are both consistent with sustained active thymic output. However, they do not discriminate the repertoire of individual T cell subtypes and, more specifically, do not show whether new TN cells were being generated. To address this point, we FACS-sorted 4 T cell subtypes (TN, TSCM, TCM, TEM) from one HD control as well as from two patients, one with high (P10) and one with low proportion of precursors T cells (P8) at two time points each after loss of engineered HSC, and collected by high throughput sequencing a total of 1193 TCR rearrangements (Supplementary Data 1). Our assumptions were the following: (a) if the thymus of these individuals was active, we would expect to detect new TCR rearrangements in TN over time and (b) if new short-lived TN were indeed continuously produced, we should observe high diversity of the TCR repertoire in this population. As shown in Fig. 3B we detected a wide diversity of TCR rearrangements in all T cell subpopulations. In P8, a lower range of VDJ recombination in TN was observed compared with P10 and the HD control, which again would be consistent with reduced thymic activity (Fig. 3C). The quantification of TCR diversity was in the range of the HD control for both patients and in all populations (Fig. 3D) including TN cells (Fig. 3E). When tracking TCR rearrangements in TN cells overtime we observed that most of them were detected only at individual time points, suggesting ongoing production of new TN cells by the thymus (Supplementary Fig. 4). Because sampling biases could affect the interpretation of longitudinal tracking of individual TCR clones, we first investigated the level of sharing of identical TCRs at each time point among the four subpopulations analysed. As shown on the network plots of Fig. 3F, sharing of identical TCR was generally low among subtypes with highest level detected between TCM and TEM in line with the biology of immunological memory and the long-term survival of these populations. As a further biological validation to our findings, in P8 we could observe, particularly at the latest time point, a substantially higher sharing of TCRs across all subtypes as compared to P10 (Supplementary Fig. 5), a result which again aligns with the progressive loss of thymic output in this individual. We then quantified the level of TCR recaptures overtime in all populations under the hypothesis that a continuous generation of new rearrangements should be reflected by low re-capture of identical TCR in TN at multiple time points. As shown in Fig. 3G, <5% of the TCRs detected in TN cells of one time point were recaptured in a second time point testifying continuous production of precursor cells with new TCR rearrangements by the thymus. Overall, the results of immunophenotypic, functional and molecular assays all lead to the conclusion that long-term de novo production of T cells by the thymus is preserved in these patients despite the lack of persistence of engineered HSC and is likely maintained by a population of vector-positive LtLP.

### Clonal tracking of T-cell subpopulations by IS analysis

**Fig. 4:**
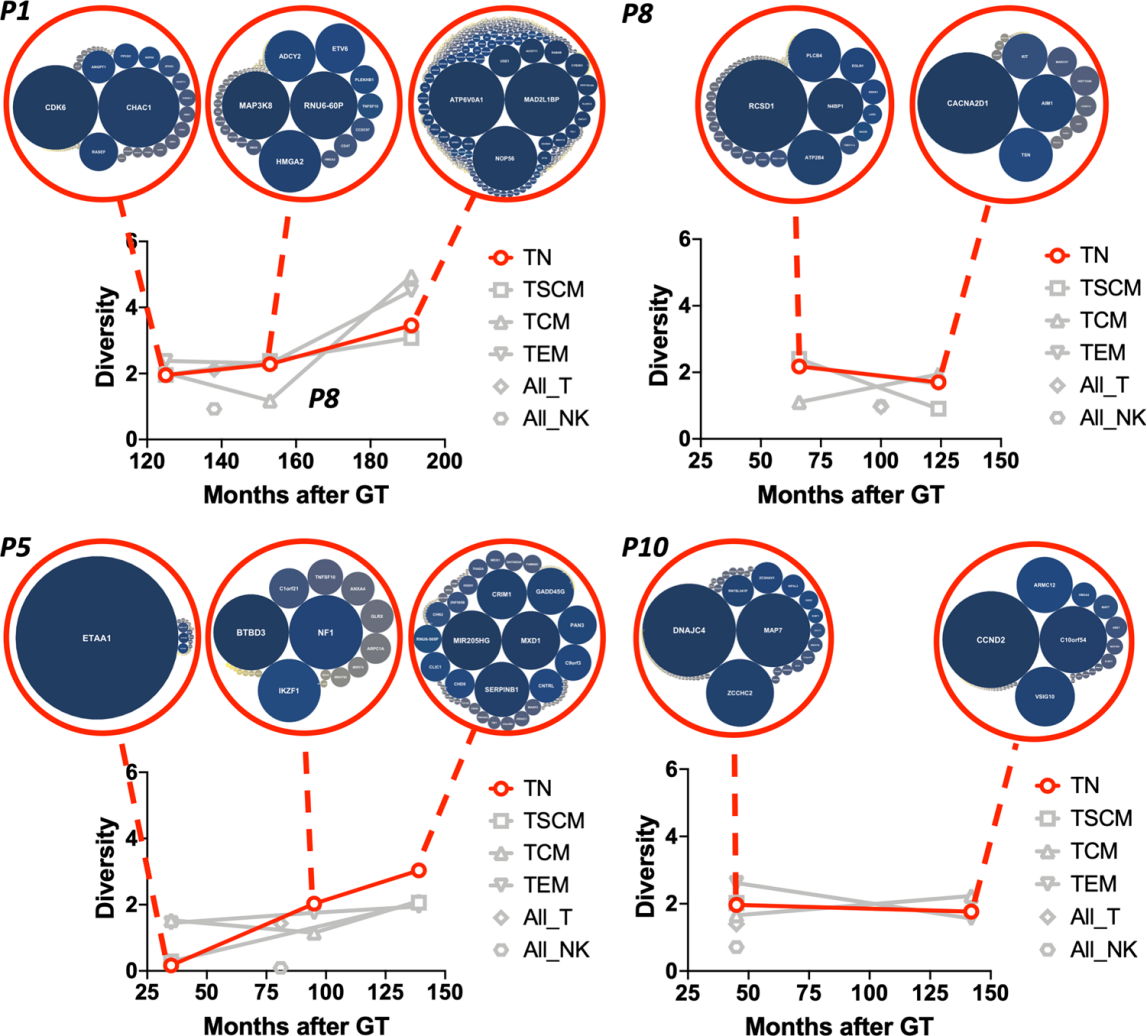
Integration sites diversity in naïve T cells and other T-cell types overtime. Plots showing Shannon Diversity Index of IS overtime in TN (red lines) and in the other T-cell subtypes (grey lines). (bubble) For TN at each timepoint analysed red circles contain bubble plots of clones contributing >0.01% to the total population. Dimension of the bubble is proportional to the size of the clone. The name of the gene closest to the relative IS is reported inside the bubble. Patients shown are the ones from which multiple timepoints were available (the single timepoint available from patient 6 is shown in Supplementary Fig. 7).

As a by-product of retroviral-mediated gene-correction, the presence of semi-randomly integrated vector sequences allows tracking of LtLP activity at clonal level by means of IS analysis, using freshly produced TN as surrogate marker of output. We therefore combined linear-amplification-mediated (LAM)-PCR with high-throughput sequencing and collected and analysed 12,756 unique IS from five populations, including TN cells, in a window of 10.1–14.9 years after loss of transplanted HSC (Supplementary Table 1 and Supplementary Data 2). We first wanted to assess whether the existence of LtLP was the result of aberrant vector-induced clonal selection or whether it represented physiological and homoeostatic survival. As shown in Supplementary Fig. 6, we occasionally detected clones contributing to more than 20% of individual T cell subtypes sampled at individual time points, as expected from physiological T cell dynamics combined with sampling biases. However, when looking at the longitudinal trend, clonal diversity remained stable in all T cell subtypes including naïve T cells (Fig 4, Supplementary Fig. 7 and Supplementary Table 4). Notably, despite a drop of circulating naïve T cells from around 100 months post GT, P8 also maintained a substantial polyclonal profile in the TN population up to the latest follow up. We then looked for IS skewing towards specific genomic loci as a marker of IS-driven clonal selection. As shown in the Supplementary Figs. 8–12, IS sites were widely distributed across multiple genes and we did not detect any evidence of selection for proto-oncogenic regions in any of the T cell subpopulations and time points analysed. Interestingly, we observed that IS of TN cells were located preferentially in regions involving gene categories such as “Leucocyte activation”, “Immune effector process” and “T-cell activation” (Supplementary Fig. 13). Because these loci were not preferentially targeted upon CD34+ cells transduction^20,21^ and because we do not have evidence for aberrant clonal selection, we can speculate that this could be the result of a physiological positive-selection for clones carrying the vector in genomic locations allowing for optimal transgene expression upon T cell differentiation/maturation.

**Fig. 5:**
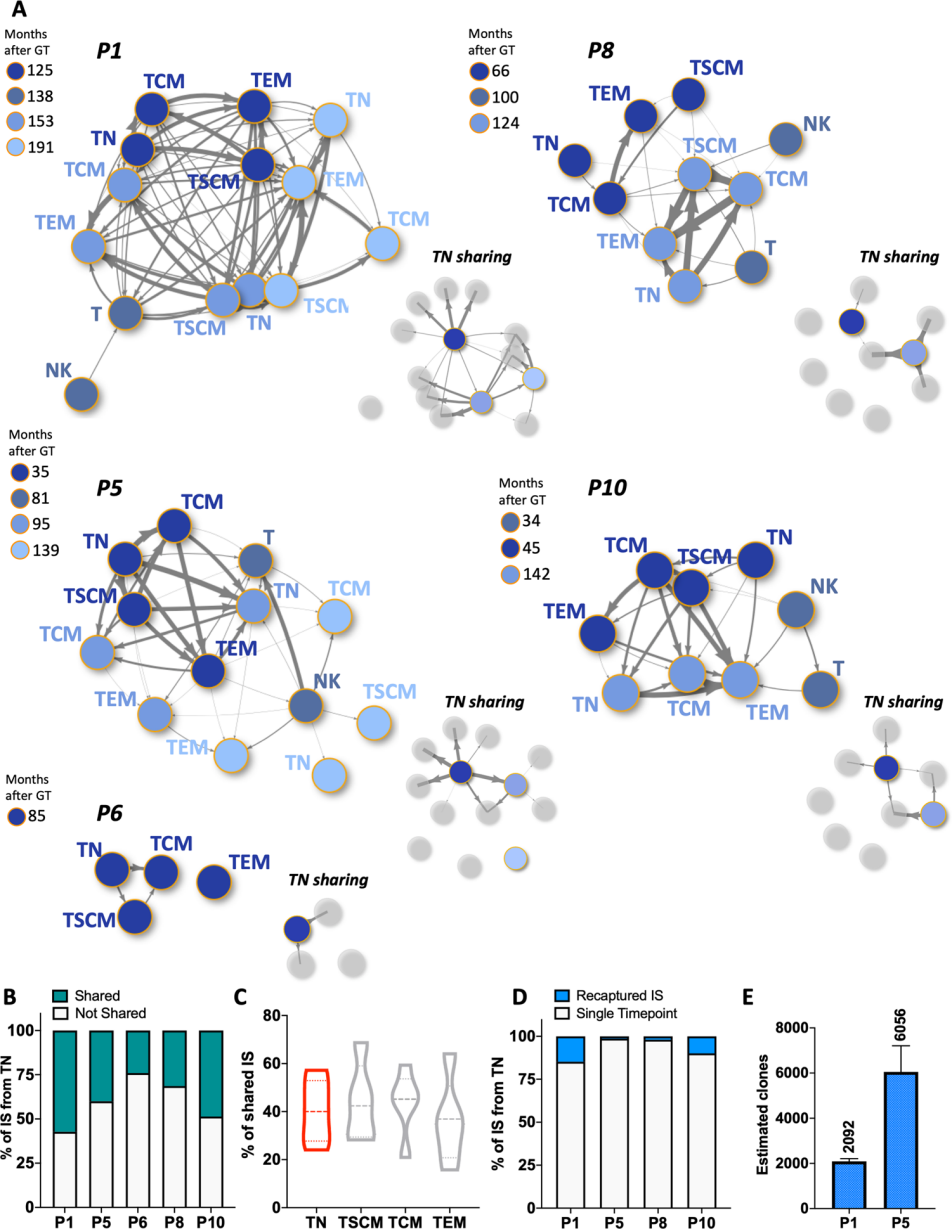
Sharing and recapture of integrations sites across T-cell subpopulations and timepoints. **A** Network plots showing significant sharing of IS among the T-cell subtypes and NK cells (data described in Fig. 5) from each patient. Each node represents a sample and it is coloured according to the timepoint after GT. The thickness of the arrows is proportional to the value of positive Pearson correlation coefficients calculated on the basis of IS sharing between pairs of samples (ranges of values are displayed on the heatmaps of Supplementary Fig. 14). For each patient two plots are shown: the networks on the left display positive correlations among all samples while the network on the right (TN sharing) highlights correlations between TN cells (nodes in scale of blue) and the rest of the subtypes (nodes in grey). **B** Stacked bars showing in green the fraction (%) of IS from TN of all timepoints for each patient which were shared with at least another T cell subtype. **C** Violin plots showing the percentage of IS belonging to each T subpopulation shared with at least another T cell subtype (data from all patients and timepoint; TN data highlighted in red). **D** Stacked bars showing in blue the fraction of IS re-captured across multiple timepoints within the TN populations of each patient (a more detailed view of re-captured IS from TN is displayed on the heatmaps of Supplementary Fig. 15). **E** Clonal abundance (number of clones in circulation) of long-term lymphoid progenitor estimated in P1 and P5 on the basis of size of IS datasets at each timepoint and re-capture probabilities overtime of IS within the TN compartment (values derived from the Mh Chao loglinear model for capture-recapture and relative standard errors as reported Supplementary Table 5).

We then looked at the presence of identical IS among different T cell subtypes to confirm that newly produced TN cells are capable of physiologically differentiating into memory subtypes in vivo. As a confirmation of the biological relevance of our data, IS sharing was high and consistent across samples collected, sorted, analysed and sequenced independently (Fig. 5A and Supplementary Fig. 14). We detected a high number of IS shared between naïve T cells and memory subsets in all patients (from 24.1% to 57.2% of IS isolated from TN of each individual) suggestive of ongoing in vivo TN differentiation (Fig. 5B). A similar range of IS sharing was detected in all subpopulations (Fig. 5C), a sign of significant clonal relationship among the T-cell subpopulations and the existence of active common progenitors. When comparing IS data with the TCR profiling, it is notable that TN shared the least number of TCR rearrangements with other subtypes at other time points but at the same time they shared equal number of IS. This suggests that individual LtLP clones generate TN with identical IS to previously generated memory cells but with new and different TCR specificities. To provide more formal evidence that individual LtLP clones are sustaining de novo T cell production over many years we used longitudinal data collected from TN cells and interrogated our dataset for detection of identical IS overtime. As shown in Fig. 5D and Supplementary Fig. 15, in all four patients from which we had T cells analysed at multiple timepoints, a fraction of IS in TN (1.3–14.8%) could be recaptured overtime in a window of up to 10.1 years from one collection to another. In the two patients where analysis could be performed at more than two time points, we used IS recapture probability to estimate the number of active engineered LtLP clones (Supplementary Table 5). In these individuals, T cell production is maintained by approximately 2000–6000 individual engineered ltLP (Fig. 5E).

### Immunophenotypic and molecular analysis of vector-positive NK cells

The fact that NK cell subset is the only other immune cell subset that still carries integrated vector copies raised the intriguing possibility that T and NK cells of these patients could share a common LtLP with dual potential. Indeed, in normal circumstances NK cells are short-lived lymphocytes, with a half-life of approximately 2 weeks, except for memory or adaptive NK cells. Memory NK cells are commonly identified by the expression of CD94/NKG2C. They are mainly observed in response to human cytomegalovirus and they can be detected months or even years after the infection^22–26^. In humans, at least five different maturation stages of NK cells have been reported identifiable on the basis of cell surface marker expression^27,28^.

**Fig. 6:**
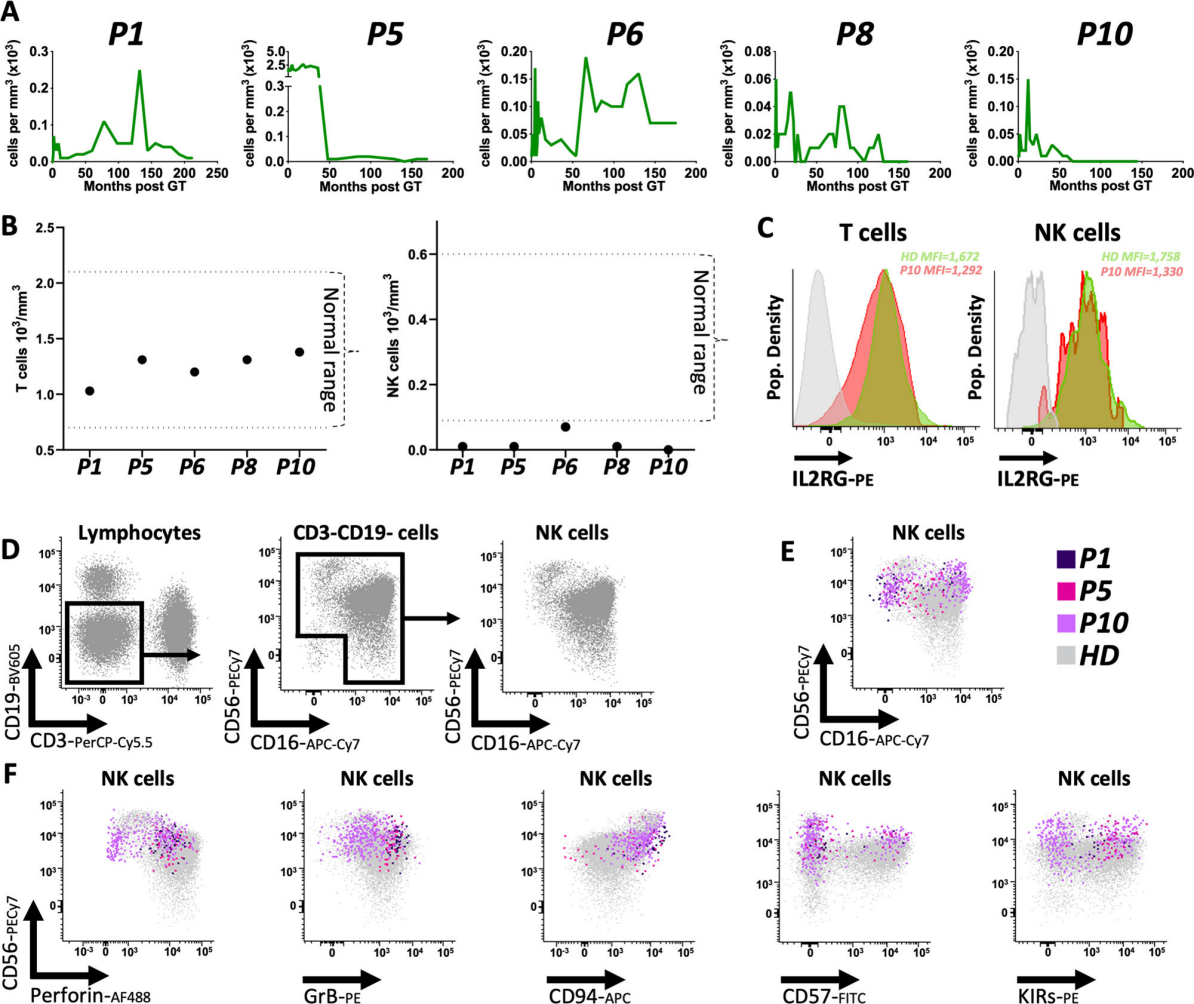
Analysis of peripheral blood contribution and immunophenotype of NK cells. **A** Absolute counts of NK cells overtime in P1, P5, P6, P8 and P10. **B** Comparison of absolute counts of T (left plot) and NK (right plot) cells in patients (black dots) as measured at the latest available timepoint vs. normal ranges measured in healthy age matched individuals (dotted lines). **C** FACS plots showing the γchain expression in T cells and NK cells in patient 10 (red) and a healthy donor (green). Isotype control is shown in grey. **D** FACS plots showing the gating scheme for the identification of NK cells within the lymphocyte population of a healthy donor used as reference. **E** Overlayed FACS plot showing the expression of CD56 and CD16 on NK cells in HD, PT1, P5 and P10. **F** Comparison of expression of Perforin, Granzyme B (GrB), CD94, CD57 and Killer-cells immunoglobulin-like receptors (KIRs) between a representative HD vs. P1, P5 and P10 inside the NK cells compartment.

Differently from what observed for T cells, the absolute number of CD56+ NK cells detected in the peripheral blood of our patient was variable overtime remaining constantly below normal levels up to the latest timepoint analysed (Fig. 6A, B). Notably however, all NK cells displayed expression of the IL2RG transgene at levels similar to T cells and to the healthy donor control (Fig. 6C). To confirm the presence of bona fide NK cells (Fig. 6D), we performed a comprehensive immunophenotyping (Supplementary Table 6) analysing the expression of CD56, CD16, Perforin, Granzyme B, CD94, CD57, KIRs, NKG2C and NKG2A in three patients and healthy donor controls (Fig. 6E, F and Supplementary Fig. 16A, B). The proportion of CD3−CD19−CD56+ and/or CD16+ true NK cells in our patients over the total lymphocyte population was low (0.2–1.2%) compared to healthy controls (8–29%) (Supplementary Fig. 16A). These data are in accordance with the VCN measurements in NK cells shown in Fig. 1C and the low absolute numbers of circulating NK cells shown in Fig. 6A, B. As for the T cells, a key question was whether these NK cells were long-lived memory cells or whether instead we could also detect bona fide NK short-living precursors suggesting that a de novo NK production is occurring. Despite the low number of cells available for this analysis (particularly in P1), CD56^bright^ NK precursors^27^ were detectable in the two of the three patients where enough events could be captured (7.7–9.9% of total NK cells) although the mean fluoresce intensity (MFI) of CD56 within this subset was slightly lower (mean = 29,616) as compared to the one measured in healthy donors used as controls (mean = 39,696) (Fig. 6E and Supplementary Fig. 16A). In order to further assess if and how many of these NK cells could be long living adaptive/memory NK cells, we also analysed the expression of NKG2C and NKG2A in P10. Very few NK cells displayed a phenotype compatible with memory/adaptive NK cells (NKG2C^+^ NKG2A^−^)^29^ (Supplementary Fig. 16B) although this definition is still contentious^30^. Also, a subset of NK cells expressed CD57, a marker generally associated with mature NK cells^23^, although it should be noted that the percentage of CD57+ cells in our patients was lower (28–30%) as compared to healthy donors (39–68%) (Fig. 6E and Supplementary Fig. 16B). This is also different from the situation described in patients with GATA2 mutations who appear to maintain an aberrant population of long living memory/adaptive NK cells^31^.

**Fig. 7:**
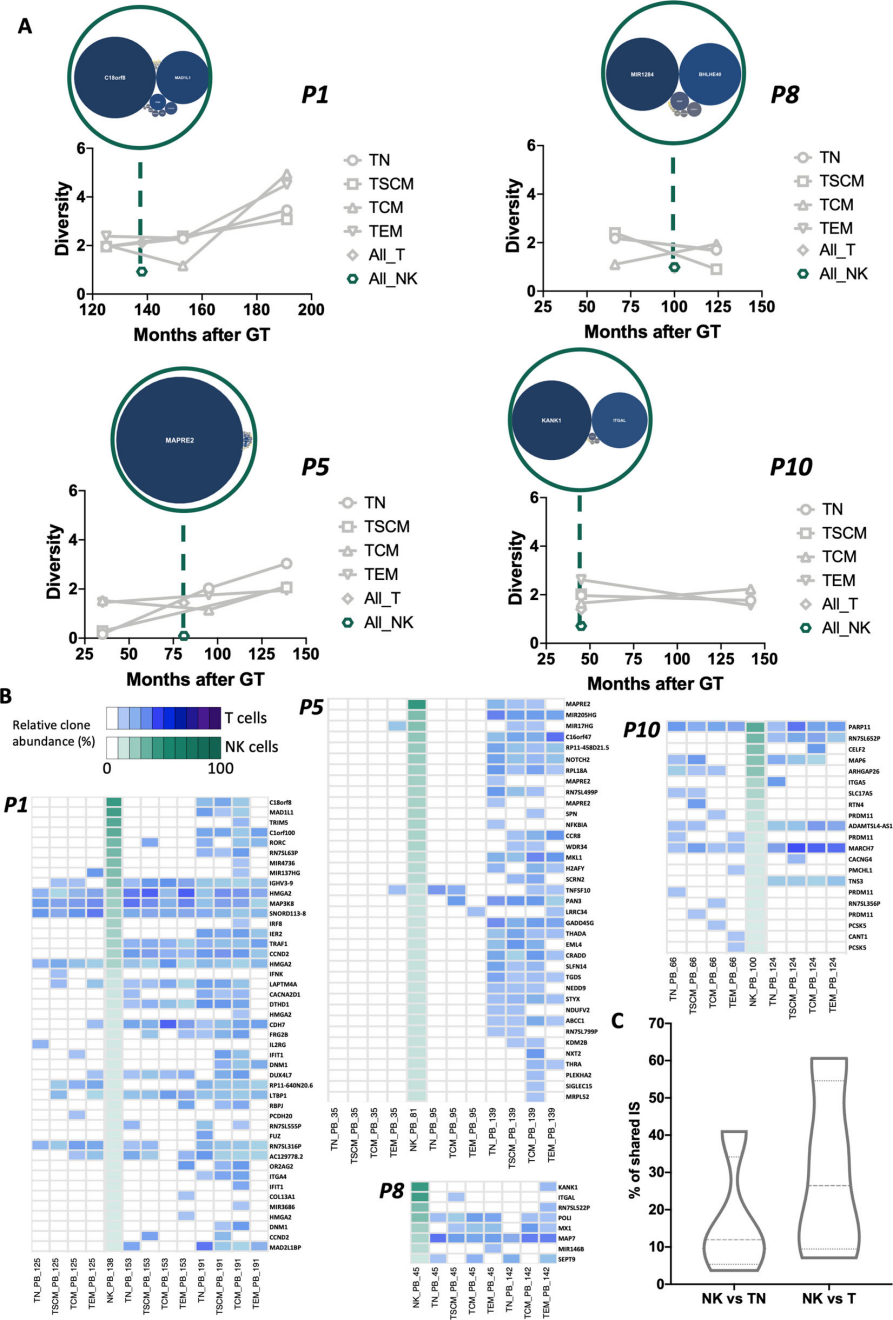
Analysis of integration sites in NK cells. **A** Plots showing Shannon Diversity Index of IS overtime in NK cells (green dots) and in the other T-cell subtypes (grey lines). At each timepoint analysed green circles contain bubble plots of clones contributing >0.01% to the total NK population. Dimension of the bubble is proportional to the size of the clone. The name of the gene closest to the relative IS is reported inside the bubble. **B** Heatmaps displaying integration sites shared between NK cells and T cell subtypes in each patient. Each row is named by the name of the closest gene while each column is labelled by sample and timepoint (number of months after GT). Intensity of green (NK) or blue (T-cell subtypes) colours is proportional to the abundance of each integration site (white = not detected). **C** Violin plots showing the percentage of NK IS shared with at least one TN (NK vs. TN) or T cell-subtype (NK vs. T) in all patients.

Having shown that a de novo NK production might be taking place, we performed molecular tracking analysis under the assumption that if NK cells originate from the same LP progenitor clones as T cells, we should be able to detect identical IS between the two subsets. To test this hypothesis, we collected and analysed 651 IS from FACS-sorted CD3−CD56+NK cells (Supplementary Tables 2 and 3). The amount and diversity of IS in NK cells were both lower as compared to IS collected from T cells (Fig. 5E), as expected from the low proportion of this lymphocyte population in the circulation of these patients when compared to T cells and to normal ranges for NK cell counts (Figs. 6B and 7A). However, and despite the limited sampling capability, we were still able to detect shared integrations between NK cells and distinct T cell subtypes (Fig. 7B and Supplementary Fig. 16C). To rule out the effect of cross-contaminations among samples we first quantified the level of shared IS between NK and TN or all T cells subpopulations which have been isolated from different time points and sequenced independently. Strikingly, we found that up to 41% of IS detected in NK cells were shared with independently analysed and sequenced TN. These numbers reach up to 60.7% when considering all T cell subpopulations from independent timepoints (Fig. 7C) and 90.2% when comparing with whole T cells samples analysed at the same timepoint (Supplementary Fig. 16C), strongly suggesting that NK and T cells are being produced de novo by a common LtLP population with dual T/NK potential.

### Analysis of ISs in proximity of proto-oncogenes

**Fig. 8:**
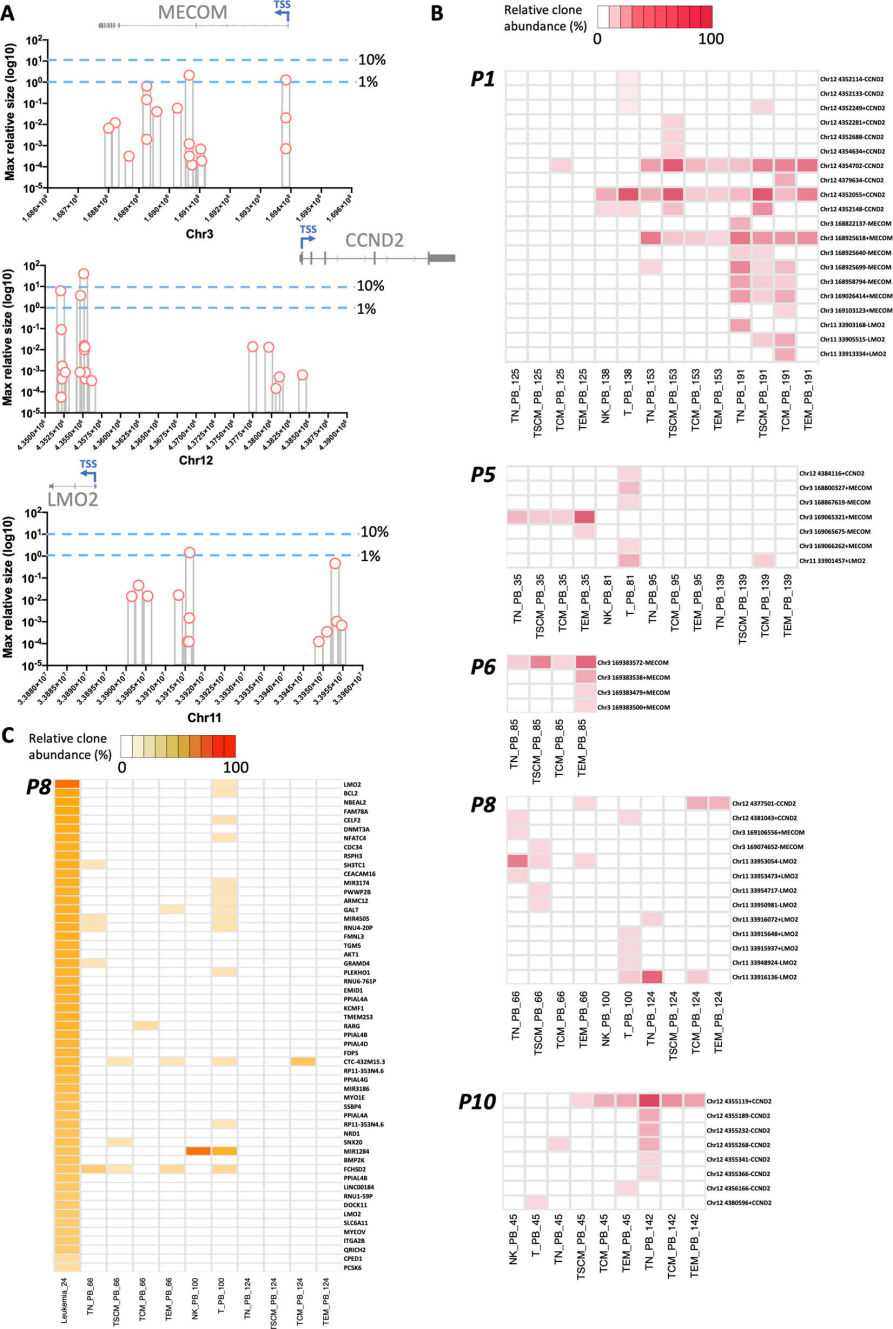
Analysis of IS in proximity of proto-oncogenes associated to insertional mutagenesis. **A** Distribution of IS in proximity in LMO2, MECOM and CCDN2 loci. In each plot the *x*-axis displays chromosomal coordinates (bp) while the *y*-axis the IS abundance (log10 of percentage) calculated within the population and timepoint where it has been observed. The light blue dotted lines show the 1% and 10% abundance thresholds. The transcription start sites (TSS), exons (grey boxes) and introns (grey lines) of each gene are shown on top of each plot in their respective chromosomal localisation. **B** Heatmaps displaying the detection of these integration sites in each patient across each sample/timepoint. Each row is named by the locus of the IS and the name of the closest gene while each column is labelled by sample and timepoint (number of months after GT). Intensity of red is proportional to the abundance of each integration site (white = not detected). **C** Heatmap showing the IS collected in the peripheral blood (PB) of P8 during leukaemia at 24 months after GT (first column) and their detection overtime. Each row is named by the name of the closest gene while each column is labelled by sample and timepoint (number of months after GT). The first row displays the LMO2 integration associated to the insertional mutagenesis event while the rest of the rows show other by-stander IS collected from PB at 24 months after GT. Intensity of orange is proportional to the abundance of each integration site (white = not detected).

As reported above, one of the patients in our trial (P8) developed a T-ALL associated to the integration of the gamma retroviral vector to the proto-oncogene LMO2 (LIM domain only 2) (1). Insertions next to other proto-oncogenic sites (CCND2, MECOM) also resulted in malignant transformation in SCIDX1 patients in a similar GT trial conducted at Necker Hospital in France (2). Of note, the first patient of our trial (P1) was treated 19 years ago, making this study the longest GT follow-up available to date. This unique clinical setting provides the opportunity to understand the long-term effects of proto-oncogenic IS. We therefore investigated the presence and the relative frequency of clones that have integrations next to the main three proto-oncogenes (LMO2, CCND2 and MECOM) that previously were involved in insertional mutagenesis events (Fig. 8A). Notably, despite the fact that we could detect a total of 52 IS in these loci, all integrations analysed contributed to <10% of the total clonal composition of each population, apart from the one clone with one IS in the CCND2 gene whose maximum relative contribution was 40% but only in one T cell subtype (TN) and at one time point (142 months after GT). Indeed, as shown in Fig. 8B some of these proto-oncogenic integrations persisted overtime but we never observed a progressive or stable expansion of any of the relative clones even over a time window spanning several years. To test whether in P8 the original leukaemic clone had reappeared and/or had contributed substantially to the survival of LtLP, we analysed a peripheral blood sample that was taken from P8 at the time of T-ALL (24 months) and compared it to samples that were taken after this time point (Fig. 8C). Confirming the accuracy of the IS quantification method used in this study, at the time of T-ALL, the IS in the LMO2 gene locus was vastly overrepresented in this patient’s peripheral blood as compared to the other IS (relative abundance = 90.8%). After 24 months this clone was not detected anymore, with the exception of a measurement in T cells at 100 months after GT which however was in the range of potential sequencing noise (relative abundance = 0.0001%). Altogether these data suggest that LtLP are physiologically maintained in these individuals and that their long-term survival is not dependent on the effects of insertional mutagenesis in proto-oncogenic loci.

## Discussion

Here we have shown that de novo T/NK production from transplanted HSPC can be maintained in humans for many years in the absence of engraftment of HSC. The absence of genetically engineered HSC in these individuals is inferred on the basis of the consistent lack of vector-positive circulating myeloid and B cells and is consistent with the fact that no myelosuppressive bone marrow conditioning was administered. The high number of detected IS and clones contributing to de novo T cell production at later time points (2000–6000) is not consistent with the notion that there is residual activity of a few multipotent HSC. Because we could not here observe a sustained output of vector positive granulocytes or monocytes, the results of this study would also indirectly suggest that human myeloid-biased HSPC are not capable of long-term survival independently from the output of multipotent HSC. This is in line with our previous study where we found no indication of long-term myeloid-biased clonal production either at the mature or at the stem/progenitor levels of haematopoiesis^8^. Conversely long-term lymphoid-biased production was clearly detected up to the multi-lymphoid progenitor level. This current work provides formal evidence that substantiates these predictions. The absence of vector-positive B cells indicates that the LtLP active in these individuals are at a differentiation stage that follows a segregation to NK/T cell fate. One working hypothesis is that these cells might resemble an early T progenitor stage previously immunophenotypically defined as CD34+CD38+CD7+ cells^1,32,33^.

The anatomic location of the LtLP identified in this study is also not clear as we are unable to sample bone marrow or thymic biopsies from these patients, although we favour the hypothesis that they are located in the thymus. Recent studies have shown that there is a significant clonal restriction in the thymus during T cell development and that very few progenitor clones can generate T cells with highly diverse TCR repertoire^34^. This suggests that thymopoiesis might be at least partially self-sustainable independently of constant progenitor influx from bona fide HSCs for the generation of fully functional naïve T cells.

It has previously been shown that the human thymus at least during foetal development contains bipotent T/NK progenitors^35^. These progenitors, defined as CD34^bright^CD1−, were able to generate both T and NK cells when cultured in mouse foetal thymic organ cultures. The fact that the majority of NK cells in our patients had non-memory/non-adaptive phenotype supports the notion that we are observing freshly produced NK cells, contrasting with data reported in patients with GATA2 mutations, who show defects in NK CD56^bright^ production and persistence of memory/adaptive NK cells^31,36^. Of note, a high percentage (28–70%) of NK cells in our patients was CD56^dim^ CD16^−^, which is normally associated with a minor subset in the peripheral blood of healthy individuals^37^. NK cells with this phenotype are often observed in higher percentages in paediatric bone marrow and leukaemic patients^38^, as well as early after HSCT^27^. Despite their functionality being poorly understood, it has been suggested that this unconventional NK subset represent an additional or alternative stage of NK cell differentiation^27^. As in our study, a reduced percentage of CD56^dim^ CD16^+^ NK cells has been observed in SCID-X1 patients years after HSC transplant and GT treatments^39^. On the other hand, our results are apparently in contrast with data reported in non-human primates^40^ in which it has been suggested that NK cells seem to have an independent origin from other lineages, including T cells. Overall, the lack of consensus for the definition of long-lived NK cells in the literature and the limited availability of patient material for a more in-depth study of the properties of these cells prevent us at this stage from reaching a definitive conclusion as to whether there is a genuine de novo production of NK cells. It is notable that circulating NK cells never (or only transiently) achieve normal levels in our patients compared to healthy controls. One can speculate that the level of gene correction or pattern of gene regulation achieved in this situation did not allow LtLP to support a full NK differentiation and/or that the observed output by these putative bipotent LtLP might be a secondary modality of production of NK cells compensating the lack of a more physiological NK cell output from HSC.

Lastly, there are methodological constraints that should be taken into account when interpreting the results of this study. It is also important to recognise that our analysis was performed in a unique disease and treatment background. In terms of experimental methodology, the use of rare clinical material derived from a small cohort of patients constrains the number of different assays that can be performed on each sample at each time point. As a consequence, despite clear evidence of population trends, statistical significance for intragroup variance or non-parametric comparison among two groups cannot always be reached. Moreover, sample viability and quality are not always consistent between fresh and long-term cryopreserved material potentially resulting in some differences in data recovery across time points (e.g. different number of IS that could be collected). Residual cross-contamination among FACS-sorted populations is another factor that can in part affect the interpretation of the results of high-throughput molecular studies. However, it should be noted that in our study the observed high-level sharing of IS from TN with other cell subtypes (up to 57.2%) far exceeded the potential effects of insufficient cell purity after sorting (estimated TN purity upon re-analysis of sorted material up to 95.6%). More importantly, identical and highly abundant IS were detected between samples that were collected, isolated, processed and sequenced at independent times.

In conclusion, our data provide the first formal evidence in vivo in humans that de novo production of genetically engineered T and NK cells can be physiologically maintained by a population of ltLP surviving many years after loss of transplanted HSC. Identification and exploitation of such human ltLP population may be of significant benefit in the development of next generation GT and cancer immunotherapy approaches.

## Methods

The reagents, methods and instruments used for the analyses described below changed during the course of the clinical trial, which was initiated in 2000. The methods described below are the ones currently in use in our laboratories. All changes in procedure were evaluated and validated to ensure consistency and comparability of the data.

### Study design

Sample size was constrained by limited clinical sample availability. For phenotypic characterisation and IS analyses of T cell subsets in SCIDX1 patients we collected in vivo biological material available during years 2001–2018. We aimed at analysing for all the patients at least 1 early and 1 late time point (where possible we did more). For IS analysis of NK and T cell identical integrations we acquired only 1 time point per patient, for TCR sequencing we aimed to acquire 1 early and 1 late time points, for IFN-gamma assay we analysed samples from the latest time points available. All available healthy donors’ and patients’ samples are reported in the manuscript. Technically validated results were always included to the analyses and we did not apply any exclusion criteria for outliers. For phenotypic characterisation of T and NK cells, Rainbow beads (RB) calibration was performed during the setup of the instrumentation for FACS analyses. RB acquisition was performed before each sample acquisition in order to achieve reproducible instrument setting among different experiments. For VCN evaluation, qPCR was validated for reproducibility. We run each sample in triplicate and values are reported as mean of the three triplicates. All attempts at replication were successful. The experimental design did not include allocation of samples to randomised experimental group. We analysed all the samples available during years 2001–2019. The experimental design did not include allocation to groups nor to blinding. There was no expected result prior to performing these analyses, therefore blinding tests were not applicable.

### Clinical trial

Clinical trial registration. There was no requirement at the time to upload to public registries.

Some patients with X-SCID were referred to GOSH through an existing network, in some cases patients with X-SCID were recruited at the research site and were already be known to the Investigators. Patients and their parents were approached by the Principal investigator and given a patient information leaflet, during this consultation the trial was discussed at length and patients have been given ample opportunity to ask questions. The trial was also approved by the Medicines and Healthcare products Regulatory Agency (MHRA). Once the patients were satisfied that they would like to enrol into the trial, the principal Investigator consented them and the process was formally documented. The Gene therapy advisory committee (GTAC in London) is UK national Research ethics committee dedicated for the review of GT clinical research. GTAC had complete oversight of this trial. There was no requirement at the time to upload to public registries, so this trial is not listed on clinicaltrials.gov. Biological material from all patients was collected in accordance with all the ethical regulations. All five patients from this study for whom no human leucocyte antigen identical sibling donor or suitable matched unrelated donor was available underwent gamma retroviral GT without any conditioning regimen. All patients we studied are alive. One patient (Pt8) developed T cell acute lymphoblastic leukaemia 24 months after GT for which he received treatment. He has since then remained in clinical and molecular remission. The data was initially recorded in the source notes which would be paper-based medical records. The laboratory data was recorded on a hospital-based electronic system which was easily accessible for the research team. Outcomes were carefully defined during the design of the trial based on input from clinicians in this field. The outcomes were based on clinical importance to look at the effect of IMP. The outcome measures were looked at by analysing patient data periodically throughout the duration of the trial, and if required would have been amended. The data was then transcribed by the study co-ordinator into paper-based case report forms (CRF). Data was recorded in the CRF in an ongoing basis and then subsequently verified by the study monitor.

### Characterisation and isolation of lymphoid and myeloid cells

Immunophenotyping was performed on whole blood EDTA samples and cell sorting was performed on PBMCs isolated from the whole blood by density gradient centrifugation using LymphoPrep (Sigma) after dextran sedimentation. Lists of antibodies used for immunophenotyping are reported in Supplementary Tables 6 and 7. Granulocytes were collected from the bottom of the lymphoprep tubes after centrifugation and red cell lysis was performed to remove red cell contaminants. FACS sorting of T, B, NK cells and monocytes from PBMCs was performed on FACS Aria (BD Biosciences) analysed with FlowJo software (TreeStar) using the following antibody panel: CD3/CD56/CD16/CD19/CD45RA FITC/PE/APC/PerCP (Multitest; BD Biosciences) and CD14 APC-Cy7 (BD Biosciences). When feasible, an aliquot of the sorted cells was re-run through the cell sorter to check fraction purity. Immunophenotyping of lymphocyte subsets was performed using a six colour multitest reagent (BD Biosciences) CD3 FITC, CD56/C16 PE, CD45 PerCP-Cy5.5, CD19 APC, CD4 PE-Cy7, and CD8-APC-Cy7. T cell immunophenotyping was performed using two antibody panels: a standard panel in place for routine clinical monitoring (CD45RA FITC, CD27 PE, CD45 PerCP, and CD4 or CD8 APC (BD Biosciences)) and a more comprehensive R&D panel CD3 V500 (BD Biosciences), CD95 PE (Biolegend), CD4 APC-Cy7 (BD Biosciences), CD8 PECy5 (BD Pharmigen), CD45RA V450 (BD Biosciences), CD62L APC (Biolegend)) developed to separate Naïve T cells, TSCMs, TCM, TEM and TEMRAs. This R&D panel was used to sort T cell subsets with FACS Aria or for immunophenotypic analysis of T cell subsets with Canto (after Rainbow bead calibration (Spherotech)). Raw FACS data was collected using DIVA software (BD Biosciences) and analysed with either Summit software for the clinical monitoring panel (BD Bisciences) or FlowJo for the R&D panel (TreeStar). To study the NK cell phenotype, PBMCs were stained with the following conjugated antibodies in two different tubes (information about the clones and company is provided in supplementary Table 6): CCR2-BV421, CCR7-BV510, CD19-BV605, CD45-BV650, CCR5-BV711, CD57-FITC, CD3-PerCP-Cy5.5, KIRs-Killer cell immunoglobulin-like receptors (CD158a, CD158b, CD158e and CD158i)-PE, CX3CR1 PE-Dazzle 594, CD56-PECy7, CXCR3-AF647, CD16 APC-Cy7, CD27-BV421, CD127-BV711, perforin-AF488, granzyme B-PE, CD94-APC. For the evaluation of cytoplasmic perforin and granzyme B, the Fix & Perm reagent kit (An der Grub, Vienna, Austria) was used, following manufacturer’s instructions. To evaluate the memory/adaptive phenotype, NKG2C PE-Vio615 and NKG2A BV510 were used (Supplementary Table 6). Common gamma chain expression in T and NK cells was analysed using the antibody CD132 PE (Biolegend, 338605, clone TUGh4) and the corresponding antibody isotype control (Biolegend, 400635, clone RTK4530). All samples were acquired in a calibrated and compensated LSR II flow cytometer (BD Biosciences) and analysed with Infinicyt (Cytognos SL). NK cells were identified as CD45+, CD3−, CD19−, CD56+ and/or CD16+ lymphocytes.

### DNA extraction and genome amplification

DNA extraction was performed using QIAamp DNA Blood Mini Kit (Qiagen) for samples of 10,000–5 × 10^6^ cells and MN NucleoSpin^®^ Tissue XS kit (manufacturer MN) for up to 10,000 cells, according to manufacturers’ instructions. DNA was quantified using Nanodrop One Spectrophotometer (Thermofisher). For IS analysis, a whole-genome amplification (WGA) was performed if the DNA yield after extraction was lower than 300 ng using the Qiagen Repli-G Mini Kit (Manufacturer), according to manufacturer’s instruction.

### VCN analysis

For VCN determination, primers and probes specific for gamma chain gene and ACTB housekeeping gene were used before 2012^41^ or gamma chain and Apo B probes and primers were used after 2012 (Supplementary Table 8). For ddPCR, ZEN was used instead of TAMRA as quencher for probes. All reactions were performed in triplicate (qPCR) or as single reactions (ddPCR) and included a 1-copy control and a non-template control (NTC) (DNase/RNase free water or elution buffer). qPCR reactions were performed using 100 ng DNA input and TaqMan Universal PCR master mix (Applied Biosystems) in CFX96 Touch thermal cycler (Bio-Rad). Data was analysed by CFX Manager (qPCR; BioRad). For ddPCR, 20 ng DNA was added to ddPCR supermix for probes (BioRad). Each sample was partitioned into >10,000 droplets using QX200 Auto droplet generator (BioRad) and the PCR was run using C1000 Touch thermal cycler (BioRad). Plates were then read in a QX200 plate reader and data was analysed using the QuantaSoft software.

### Vbeta spectratyping analysis

CDR3 TCR spectratyping was performed as described previously^42^. Briefly, RNA was extracted and complementary DNA (cDNA) was prepared from CD3^+^, CD4^+^, and/or CD8^+^ T cell subsets. Twenty-four Vβ-specific primers were used with a fluorescent-labelled constant region (Cβ)-specific primer to RT-PCR (reverse transcription-PCR)-amplify the CDR3 region of the TCRβ chain (Supplementary Table 11). The labelled products were loaded onto an ABI 310 Automate (Perkin-Elmer Applied Biosystems, UK), which measures the intensity of fluorescence (the amount of PCR product), and, by reference to the detection time of the internal size standards, the length of the PCR products was determined and analysed with SpA-based software^43^.

### TREC analysis

Real-time qPCR targeting a specific marker of functional T cells, the TREC, was performed as described previously^43^. Briefly, DNA was extracted from CD3+, CD4+, and/or CD8+ T cell subsets and subjected to a multiplex qPCR to amplify TRECs and the RNaseP housekeeping gene (Supplementary Table 8). By reference to standard curves, generated with a TREC-containing plasmid^44^ and dilutions of genomic DNA for the RNaseP gene, TREC numbers were calculated for each sample.

Forward and reverse primers targeting δRec-ΨJα TREC-specific sequences were used to generate a 93-bp amplicon spanning the splice junction, with the TREC probe located just downstream from the junction. The qPCR included primers and probe for an attenuated amplification of the RNase P gene *RPPH1*. The 20-μL reaction consisted of 10 μL TaqMan^®^ Fast Universal PCR Master Mix (4367846; Applied Biosystems), 0.4 μL TaqMan RNase P Vic Control Reagent (4316844; Applied Biosystems), and TREC primers and probe sequences (Applied Biosystems) located within the gene identified by accession number [NT_026437, nucleotides (forward primer) 3944229 through 3944289, and (reverse primer) 3855229 through 3855280] in the following concentrations: 8 pmol each of forward TREC primer (TGCTGACACCTCTGGTTTTTGTAA) and reverse TREC primer (GTGCCAGCTGCAGGGTTTAG), 3 pmol TREC-specific hydrolysis probe (6FAM-ATGCATAGGCACCTGC-MGB), and 5 μL of DNA eluate. Absolute qPCR was performed in an Applied Biosystems 7900 HT Real-Time PCR System in a 384-well plate (4343814; Applied Biosystems).

### IFN-gamma production assay

T cells from patients and healthy donors (HD) were FACS sorted into TN, TSCM, TCM and TEM subsets and a minimum of 10,000 cells were used for IFN-gamma assay. A list of antibodies used for this assay is reported in Supplementary Table 7. Cells were incubated in medium (RPMI) containing 10% human AB serum, 1% penicillin–streptomycin, PMA (50 ng/ml) and ionomycin (1 µg/ml) at 37 °C for 6 h. After stimulation, brefeldin A (10 µg/ml) was added to the medium, and cells were further incubated for 2 h. At the end of incubation, cells were fixed and permeabilised with BD Cytofix/Cytoperm™ for 20 min at 4 °C, and stained with FITC-conjugated IFN-gamma antibody (BD Biosciences) in BD Perm/Wash™ buffer for 30 min at 4 °C. Cells were then washed with BD Perm/Wash™ buffer, re-suspended in fixation buffer and analysed with BD FACS Aria. Data was acquired using Diva software and analysed using Flow Jo.

### TCR collection and library preparation

High-throughput sequencing of TCR was performed on TN, TSCM, TCM and TEM FACS-sorted populations as previously described^45^. Briefly, PCR was performed to amplify somatic rearrangements within TCR gamma, TCR delta and TCR beta genes (Supplementary Tables 12, 13). After the initial PCR, the products were purified via Pronex bead chemistry (Promega) and sequencing adaptors were added. These adaptors allow the sequences to bind to the flow cell in the MiSeq and also contain indices used to identify samples during sequencing analysis. Dual indexing utilises two 8-base sequences; index 1 (i7), adjacent to the P7 sequence and index 2 (i5), adjacent to the P5 sequence (Supplementary Tables 14–16). Resulting amplicons were normalised and pooled based on Qubit and Tapestation values and sequenced using Illumina MiSeq 300 v2 kit. TCR sequencing demultiplexing was performed using error aware demultiplexer (EAD) for Illumina BCL files (v1.0.3). The resulting FASTQ files were then loaded on the Vidjil (High-Throughput Analysis of V(D)J Immune Repertoire) suite (www.vidjil.org).

### Statistical and computational analyses

Unless otherwise specified, plots and statistical analyses were generated with Prism 5 (GraphPad Software). Statistical significance among groups was measured using Kruskal–Wallis test for intragroup variance, Mann–Whitney test, and unpaired *t*-test.

### High throughput TCR sequencing analyses

After TCR sequencing demultiplexing was performed using EAD for Illumina BCL files (v1.0.3). The resulting FASTQ files were then loaded on the Vidjil (high-throughput analysis of V(D)J immune repertoire) suite (www.vidjil.org). Data shown in Fig. 3b, c were generated on the basis of the TCR database in Supplementary Material 1 exported from Vidjil and filtered for rearrangements labelled with “no CD3 detected”. TCR diversity was calculated as Shannon Diversity Index through the R package Entropy (http://cran.r-project.org/web/packages/entropy/index.html).The network plots of Fig. 3f were generated using the R package visNetwork (https://cran.r-project.org/web/packages/visNetwork/index.html). Pearson correlation values for these plots were generated through the R package Hmisc (https://cran.r-project.org/web/packages/Hmisc/index.html, function rcorr, type = “pearson”).

### ISs analyses

IS identification and analysis were performed through a custom analytical pipeline extensively portrayed in previous publications^7,15^. Briefly, two rounds of linear PCR were performed (50 cycles each) to enrich for vector genome junctions using biotinylated primers specific for vector LTRs. Streptavidin-coupled magnetic beads (Invitrogen Dynabeads Kilobase Binder Kit) were added to each sample, to capture linearly amplified fragments, followed by complementary strand synthesis. Samples then underwent restriction enzyme digest using three different enzymes—MluCI, AciI or HpyCH4IV, to minimise bias and improve genome coverage. Linker cassettes were added and samples underwent two rounds of exponential amplification PCR to amplify fragments containing vector LTR and linker cassette sequences. PCR fragment size depends on the distance between the known vector sequence and the closest enzyme recognition site. To visualise amplified fragments, we used high-resolution gel electrophoresis (ElchromScientific). Final PCR products were purified with QIAQUICK PCR purification kit (QIAGEN) and DNA was quantified using Nanodrop One Spectrophotometer. Fusion PCR was used to add sequence-specific Illumina adaptors to the final PCR products. Different combinations of LTR primers and LC primers were used to differentiate between different samples.

The following is a brief summary of the computational tools used for the analysis of IS. Raw ISs data sets underwent series of different bioinformatics filtering procedures according to the type of analysis to be performed. All data sets were processed with a “collision detection filter” to univocally assign each IS to a patient and to one or more T cell subpopulations by applying a 10-fold rule for contamination identification as previously reported. A final matrix *M* was generated where each row *r* represented an individual IS while each column *c* an individual cell type/sample and time point. Each entry of *M* contained the abundance of each *r* for each *c* in terms of sequencing reads. The data shown in Figs. 4,5–7,8 were generated on the basis of the IS databases summarised in Supplementary Tables 3 and 4 attached to Supplementary Material 2. Panels of Figs. 4 and 7a were created plotting IS diversity overtime calculated as Shannon Diversity Index through the R package Entropy (http://cran.r-project.org/web/packages/entropy/index.html). Additional diversity indexes Simpson and InverseSimpson were calculated and reported together with Shannon diversity in Supplementary Table 4 through the use of the R package BiodiversityR (https://cran.r-project.org/web/packages/BiodiversityR/index.html). The “bubble” plots on top of each panel were created on the basis of the IS from TN and NK, respectively, with abundance >0.01% relative to each subpopulation and time point using the R package packcircles (https://cran.r-project.org/web/packages/packcircles/index.html). The network plots of Fig. 5a were generated using the R package visNetwork (https://cran.r-project.org/web/packages/visNetwork/index.html). The Pearson correlation values for these plots and for the ones of Supplementary Fig. 14, were generated through the R package Hmisc (https://cran.r-project.org/web/packages/Hmisc/index.html, function = rcorr, type = “pearson”). Estimation of clonal abundance and standard errors shown in Fig. 5e and Supplementary Table 5 could be calculated on the two IS datasets with three timepoints each (P1 and P5) by the conversion of *M* to a *M*(0,1) matrix of incidence and by the application to *M*(0,1) of log-linear models for closed populations through the R package Rcapture (https://cran.r-project.org/web/packages/Rcapture/index.html, function = closedp.t). The Mth Chao (LB) method was selected for visualisation in Fig. 5e being the most conservative estimation among the ones with the lowest Bayesian Information content (BIC). The heat maps of Figs. 7b, 8b, c and Supplementary Fig. 15 were generated through the R package gplots (https://cran.r-project.org/web/packages/gplots/index.html) with ramping gradients of colour palettes calculated using the following breaks for relative IS abundance (col_breaks=c(seq(0,0.0001,length=100), seq(0.00011,0.01,length = 100), seq(0.011,0.1,length = 100),seq(0.11,1,length = 100), seq(1.1,5,length = 100), seq(5.1,10,length = ), seq(10.1,20,length = 100), seq(20.1,30,length = 100), seq(30.1,50,length = 100), seq(50.1,70,length = 100), seq(70.1,100,length = 100)). Word clouds of Supplementary Figs. 8–12 and Supplementary Fig. 13 (top panel) were generated on the basis the relative incidence of the single closest gene to each IS and plotted using the online suite WordClouds (https://www.wordclouds.com). The Gene Ontology analysis of hit genes in TN shown in Supplementary Fig. 13 (bottom panel) was generated through the online suite Genomic Regions Enrichment of Annotations Tool (GREAT) (http://great.stanford.edu/public/html/) using BED data from IS coordinates, associating genomic regions with the rule of single nearest gene within 1000 kb to each IS and applying the gene annotations to these regions.

### Reporting summary

Further information on research design is available in the Nature Research Reporting Summary linked to this article.

## Supplementary information

Supplementary Information

Reporting Summary

Description of Additional Supplementary Files

Supplementary Data 1

Supplementary Data 2

## Data Availability

The authors declare that a list of all TCR rearrangements and IS data supporting the findings of this study are available within the paper’s Supplementary information (Supplementary Data 1 and 2).
