# Supplementary material for: Long-term lymphoid progenitors independently sustain naïve T and NK cell production in humans
Source: Nat Commun. 2021 Mar 12;12:1622. doi: 10.1038/s41467-021-21834-9 (PMC7954865; doi:10.1038/s41467-021-21834-9)
Supplement: Supplementary file 1 — Supplementary Information [file 41467_2021_21834_MOESM1_ESM.pdf]

FigS1

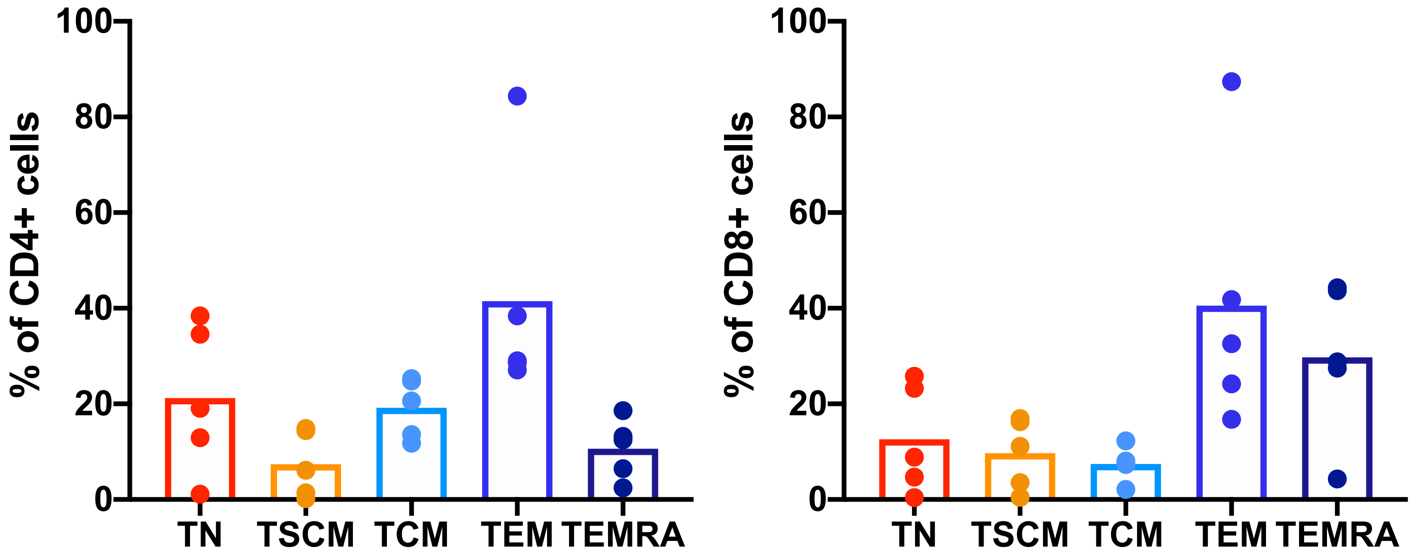

Supplementary Figure 1 Percentage of TN, TSCM, TCM, TEM and TEMRA inside the CD4+ (left panel) or CD8+ (right panel) population at latest follow up in the 5 patients analysed

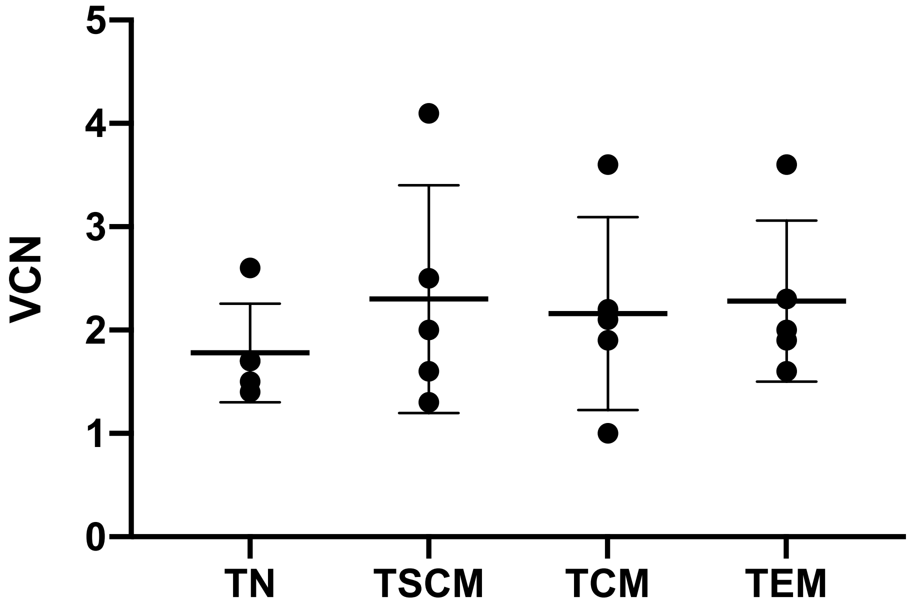

Supplementary Figure 2 Vector copy number (VCN) measured in different T cell subsets at latest available time points post GT (P1 – 17 years post; P5 – 12 years post; P6 – 7 years post; P8 – 6 years post; P10 – 11 years post). Each dot represents VCN retrieved from one patient. Mean and standard deviations are also shown for each subpopulation analysed.

FigS3

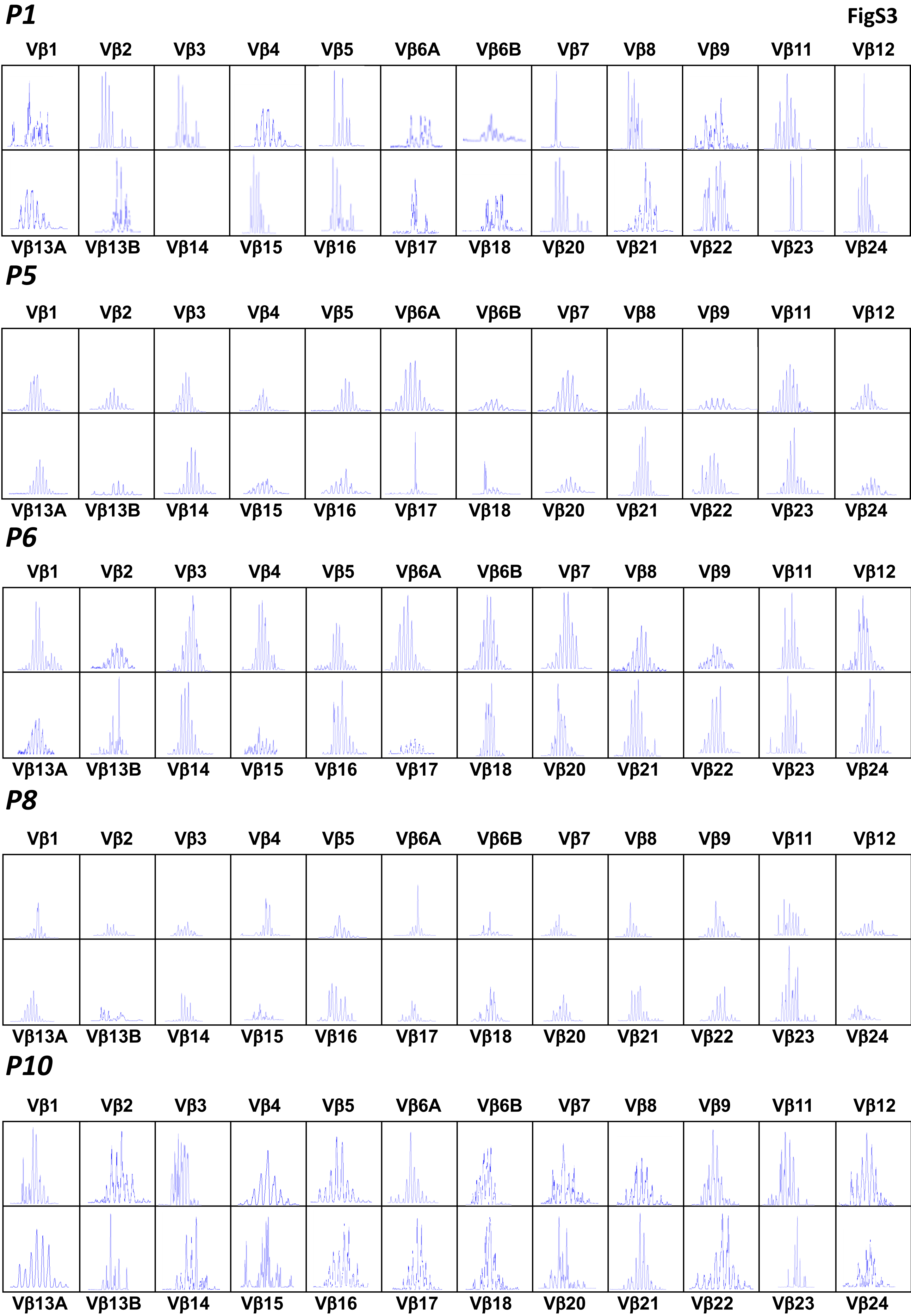
P1
Vβ1 Vβ2 Vβ3 Vβ4 Vβ5 Vβ6A Vβ6B Vβ7 Vβ8 Vβ9 Vβ11 Vβ12
Vβ13A Vβ13B Vβ14 Vβ15 Vβ16 Vβ17 Vβ18 Vβ20 Vβ21 Vβ22 Vβ23 Vβ24
P5
Vβ1 Vβ2 Vβ3 Vβ4 Vβ5 Vβ6A Vβ6B Vβ7 Vβ8 Vβ9 Vβ11 Vβ12
Vβ13A Vβ13B Vβ14 Vβ15 Vβ16 Vβ17 Vβ18 Vβ20 Vβ21 Vβ22 Vβ23 Vβ24
P6
Vβ1 Vβ2 Vβ3 Vβ4 Vβ5 Vβ6A Vβ6B Vβ7 Vβ8 Vβ9 Vβ11 Vβ12
Vβ13A Vβ13B Vβ14 Vβ15 Vβ16 Vβ17 Vβ18 Vβ20 Vβ21 Vβ22 Vβ23 Vβ24
P8
Vβ1 Vβ2 Vβ3 Vβ4 Vβ5 Vβ6A Vβ6B Vβ7 Vβ8 Vβ9 Vβ11 Vβ12
Vβ13A Vβ13B Vβ14 Vβ15 Vβ16 Vβ17 Vβ18 Vβ20 Vβ21 Vβ22 Vβ23 Vβ24
P10
Vβ1 Vβ2 Vβ3 Vβ4 Vβ5 Vβ6A Vβ6B Vβ7 Vβ8 Vβ9 Vβ11 Vβ12
Vβ13A Vβ13B Vβ14 Vβ15 Vβ16 Vβ17 Vβ18 Vβ20 Vβ21 Vβ22 Vβ23 Vβ24

Supplementary Figure 3 Vbeta profiles measured by spectratyping in all patients at their latest follow up available (from 110 months to 210 months after GT).

FigS4

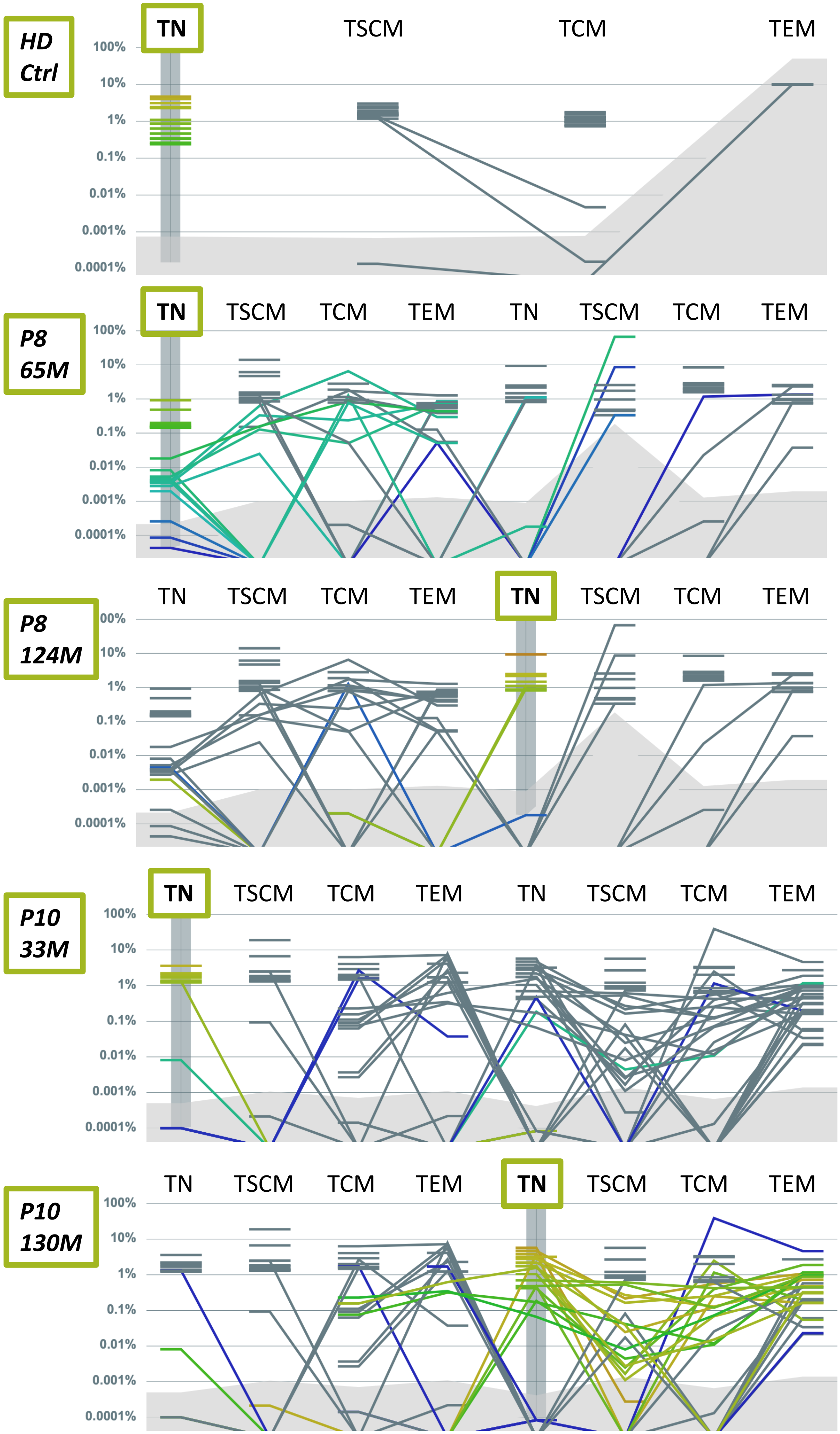
HD Ctrl
TN
TSCM
TCM
TEM
100%
10%
1%
0.1%
0.01%
0.001%
0.0001%
P8 65M
TN
TSCM
TCM
TEM
TN
TSCM
TCM
TEM
P8 124M
P10 33M
P10 130M

Supplementary Figure 4 Relative abundance and tracking of TCR rearrangements in HD and patients overtime. The y-axis displays relative abundance (log10 scale). Horizontal tiles show most abundant TCRs in each sample. Connecting lines show detection of the most abundant TCRs in each subpopulation across other samples and timepoints. The grey area displays average distribution of TCR relative abundance in each subpopulation. Data collected in TN at a given time point (on the left, M=months) are highlighted in green in the relative plot. Plots were generated using the Vidjil platform (www.vidjil.org).

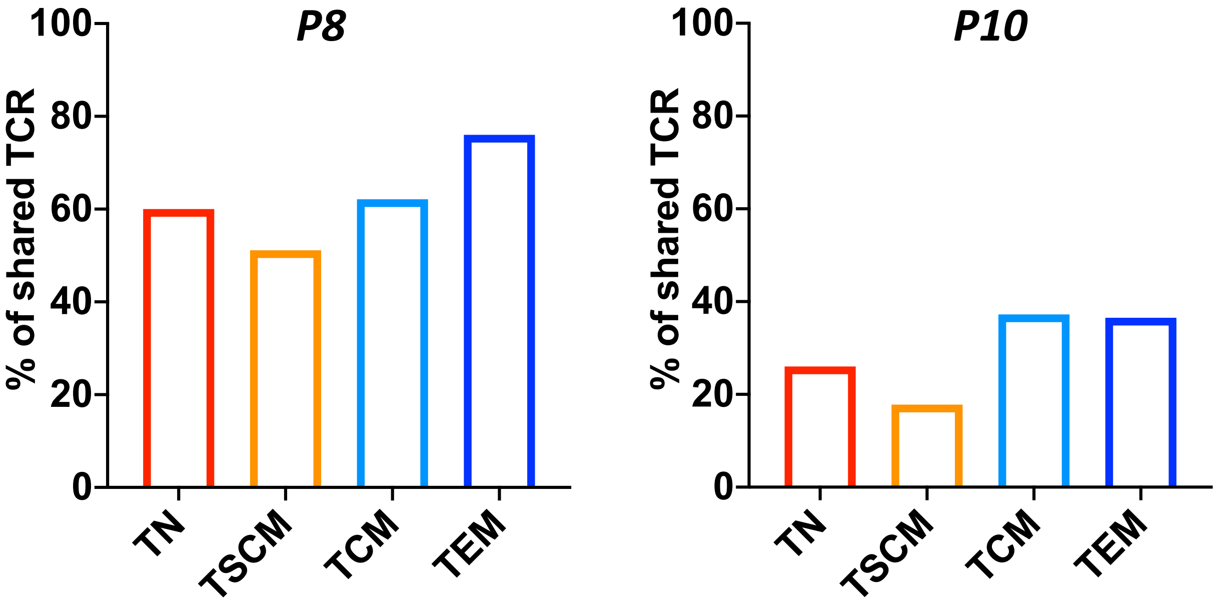

Supplementary Figure 5 Percentage of TCR rearrangements shared by each T -cell subtype with at least another T-cell subpopulation in P8 (left panel) and P10 (right panel).

FigS6

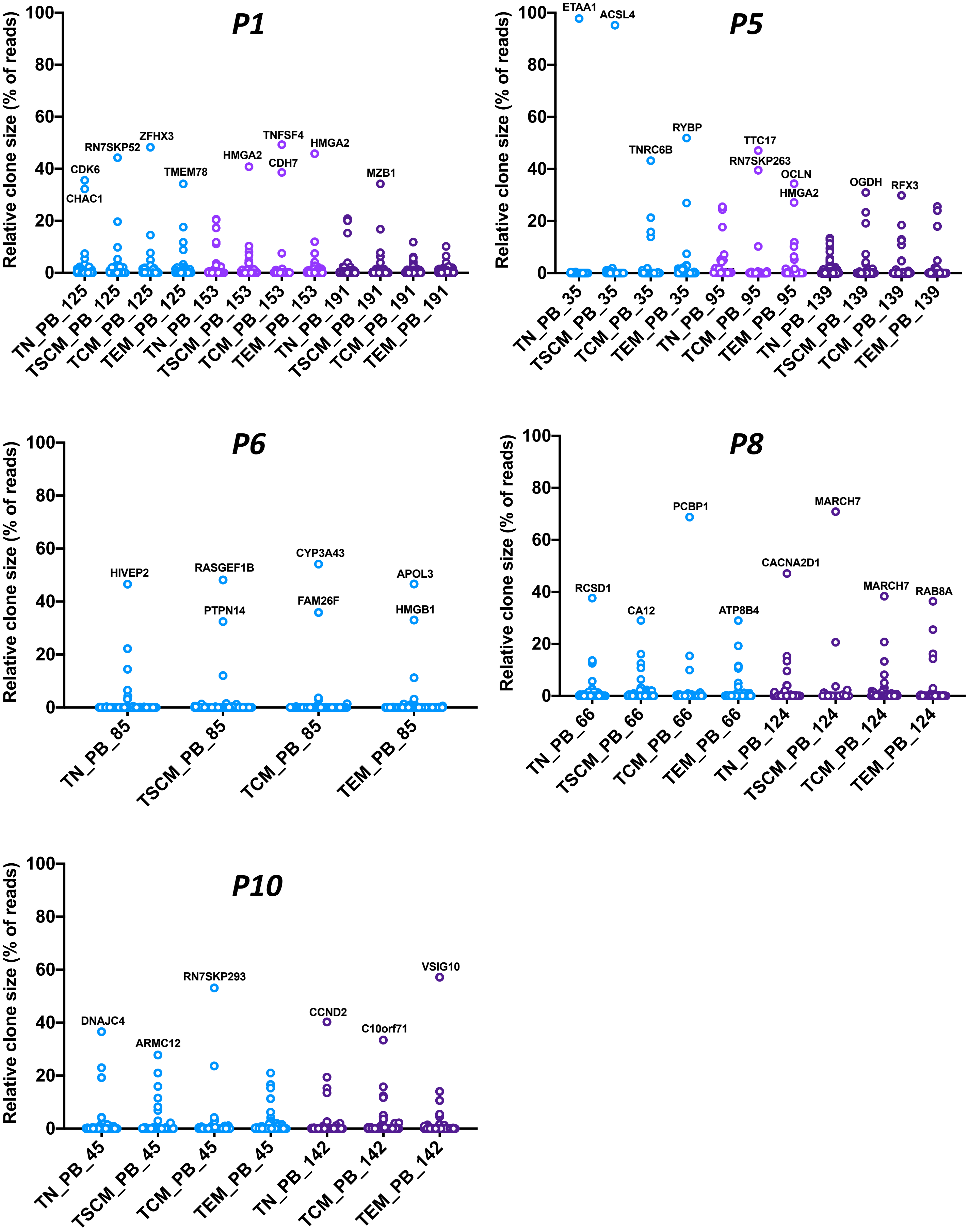

P1
Relative clone size (% of reads)
0
20
40
60
80
100
CDK6
CHAC1
RN7SKP52
ZFHX3
TMEM78
HMGA2
TNFSF4
CDH7
HMGA2
MZB1
TN_PB_125
TSCM_PB_125
TCM_PB_125
TEM_PB_125
TN_PB_153
TSCM_PB_153
TCM_PB_153
TEM_PB_153
TN_PB_191
TSCM_PB_191
TCM_PB_191
TEM_PB_191
P5
ETAA1
ACSL4
TNRC6B
RYBP
TTC17
RN7SKP263
OCLN
HMGA2
OGDH
RFX3
TN_PB_35
TSCM_PB_35
TCM_PB_35
TEM_PB_35
TN_PB_95
TCM_PB_95
TEM_PB_95
TN_PB_139
TSCM_PB_139
TCM_PB_139
TEM_PB_139
P6
HIVEP2
RASGEF1B
PTPN14
CYP3A43
FAM26F
APOL3
HMGB1
TN_PB_85
TSCM_PB_85
TCM_PB_85
TEM_PB_85
P8
RCSD1
CA12
PCBP1
ATP8B4
CACNA2D1
MARCH7
MARCH7
RAB8A
TN_PB_66
TSCM_PB_66
TCM_PB_66
TEM_PB_66
TN_PB_124
TSCM_PB_124
TCM_PB_124
TEM_PB_124
P10
DNAJC4
ARMC12
RN7SKP293
CCND2
C10orf71
VSIG10
TN_PB_45
TSCM_PB_45
TCM_PB_45
TEM_PB_45
TN_PB_142
TCM_PB_142
TEM_PB_142

Supplementary Figure 6 Relative abundance of IS in each T cell subpopulation and patient. Scale of blue is used to group samples according to timepoint of analysis. The most abundant IS (>30%) are labelled with the name of the closest gene for each sample and timepoint (PB = peripheral blood; numbers = months after GT).

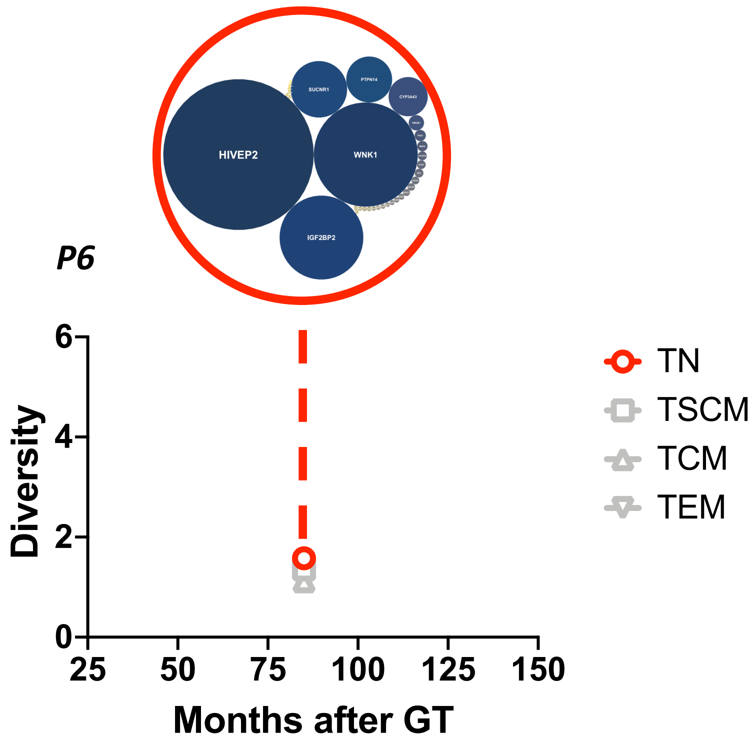

Supplementary Figure 7 Plots showing Shannon Diversity Index of IS overtime in TN (red lines) and in the other T-cell subtypes (grey lines). (bubble) For TN at each timepoint analysed red circles contain bubble plots of clones contributing >0.01% to the total population. Dimension of the bubble is proportional to the size of the clone. The name of the gene closest to the relative IS is reported inside the bubble. (single timepoint available from patient 6)

FigS8

P1

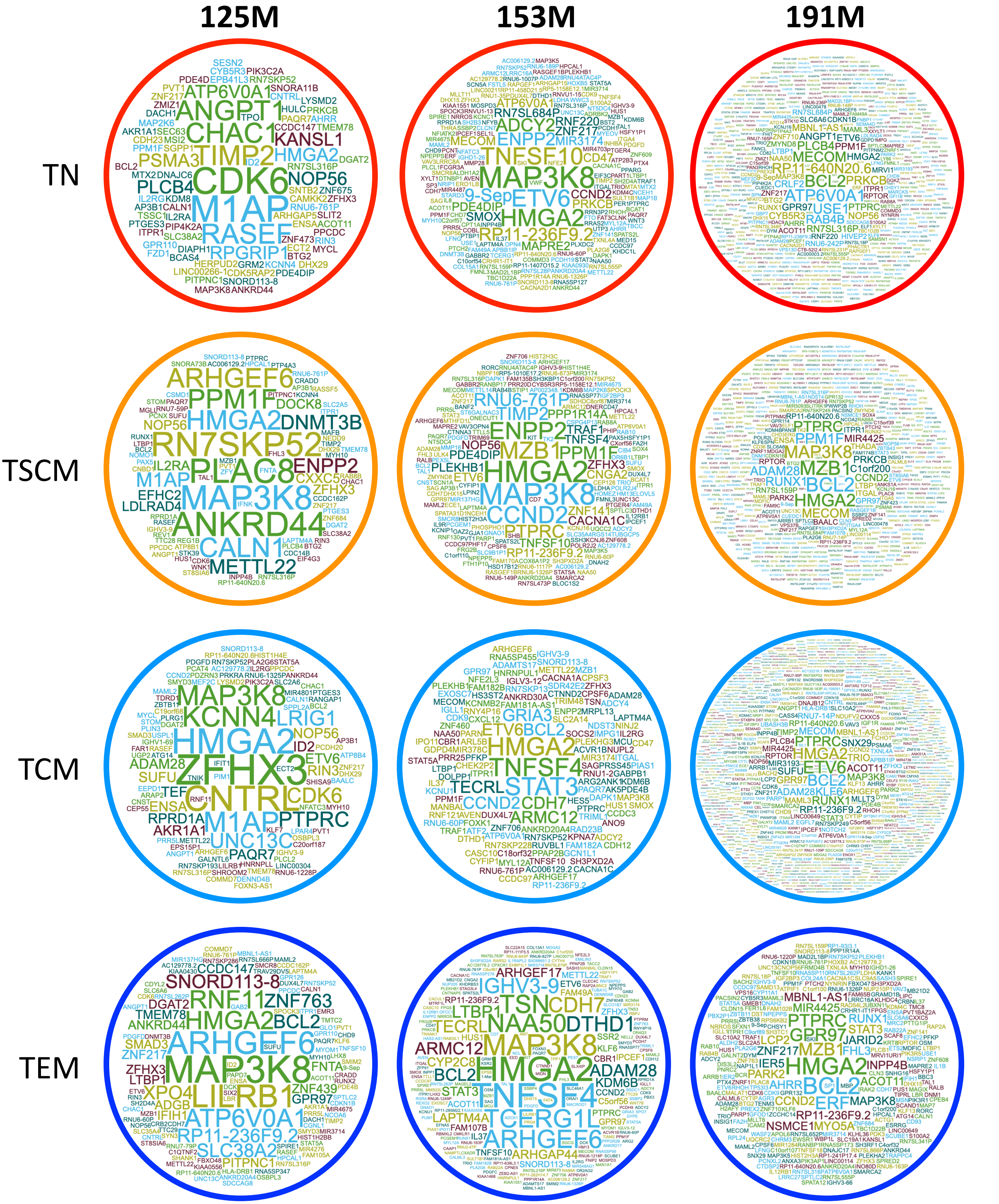

Supplementary Figure 8 Word clouds showing the genomic distribution of IS from P1 in each sample and timepoint. Relative size of each gene is proportional to the relative level of IS clustering (the bigger it is the gene name the higher it is the number of IS detected in its proximity).

FigS9

P5

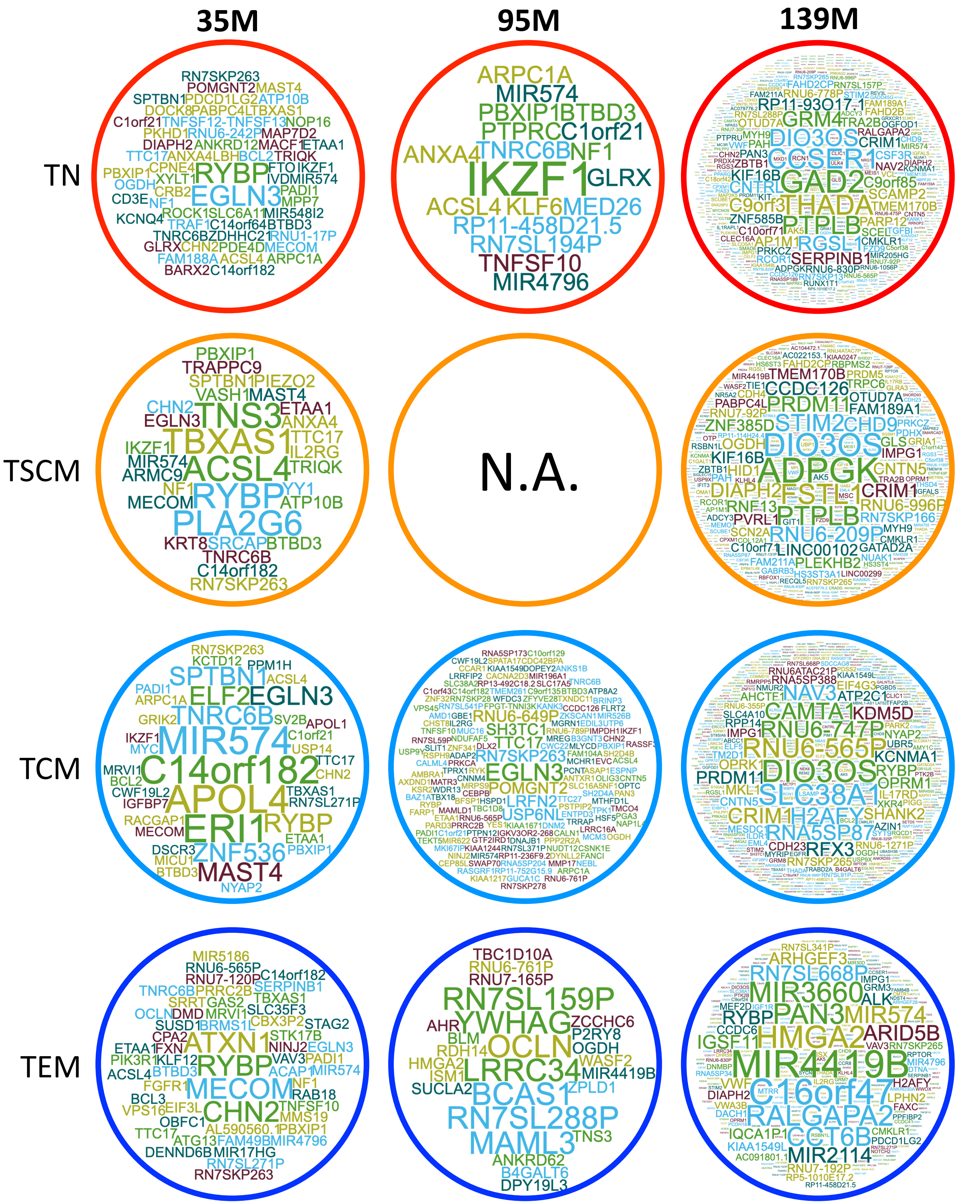

Supplementary Figure 9 Word clouds showing the genomic distribution of IS from P5 in each sample and timepoint. Relative size of each gene is proportional to the relative level of IS clustering (the bigger it is the gene name the higher it is the number of IS detected in its proximity).

FigS10

P6

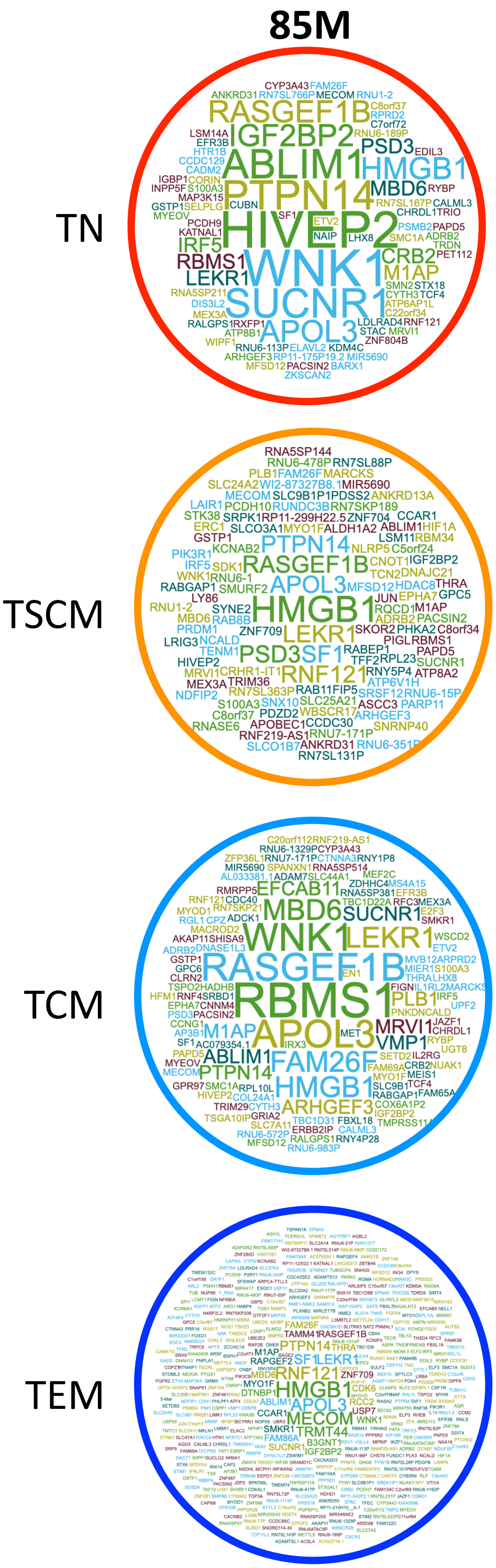
85M
TN
RASGEF1B
IGF2BP2
ABLIM1
HMGB1
PTPN14
HIVEP2
WNK1
SUCNR1
APOL3
TSCM
HMGB1
RASGEF1B
APOL3
PTPN14
LEKR1
PSD3
SF1
RNF121
TCM
WNK1
RASGEF1B
RBMS1
APOL3
MBD6
EFCAB11
LEKR1
FAM26F
HMGB1
ABLIM1
PTPN14
ARHGEF3
TEM
HMGB1
APOL3
MECOM
RNF121
PTPN14
LEKR1
TRMT44

Supplementary Figure 10 Word clouds showing the genomic distribution of IS from P6 in each sample and timepoint. Relative size of each gene is proportional to the relative level of IS clustering (the bigger it is the gene name the higher it is the number of IS detected in its proximity).

FigS11

P8

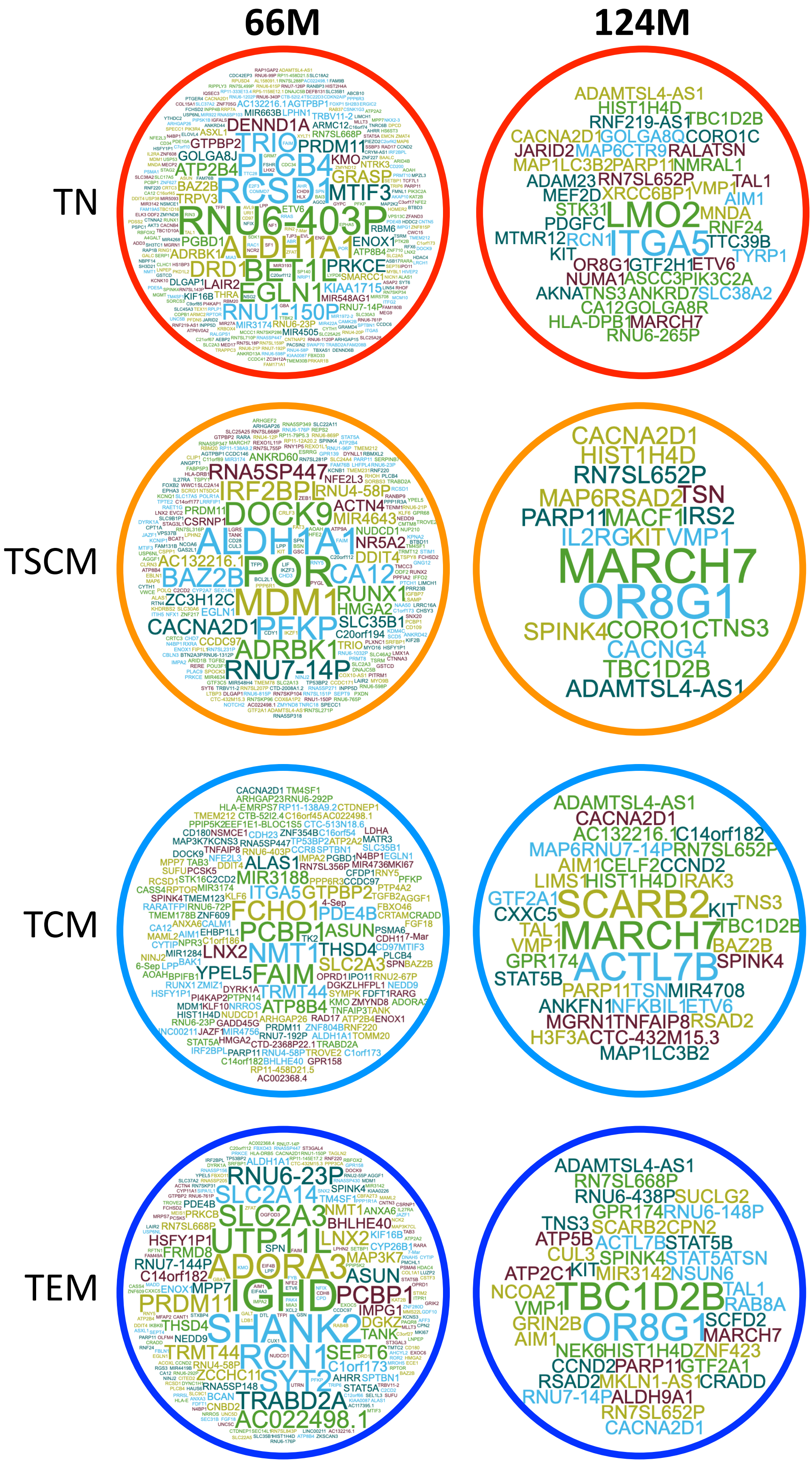

Supplementary Figure 11 Word clouds showing the genomic distribution of IS from P8 in each sample and timepoint. Relative size of each gene is proportional to the relative level of IS clustering (the bigger it is the gene name the higher it is the number of IS detected in its proximity).

FigS12

P10

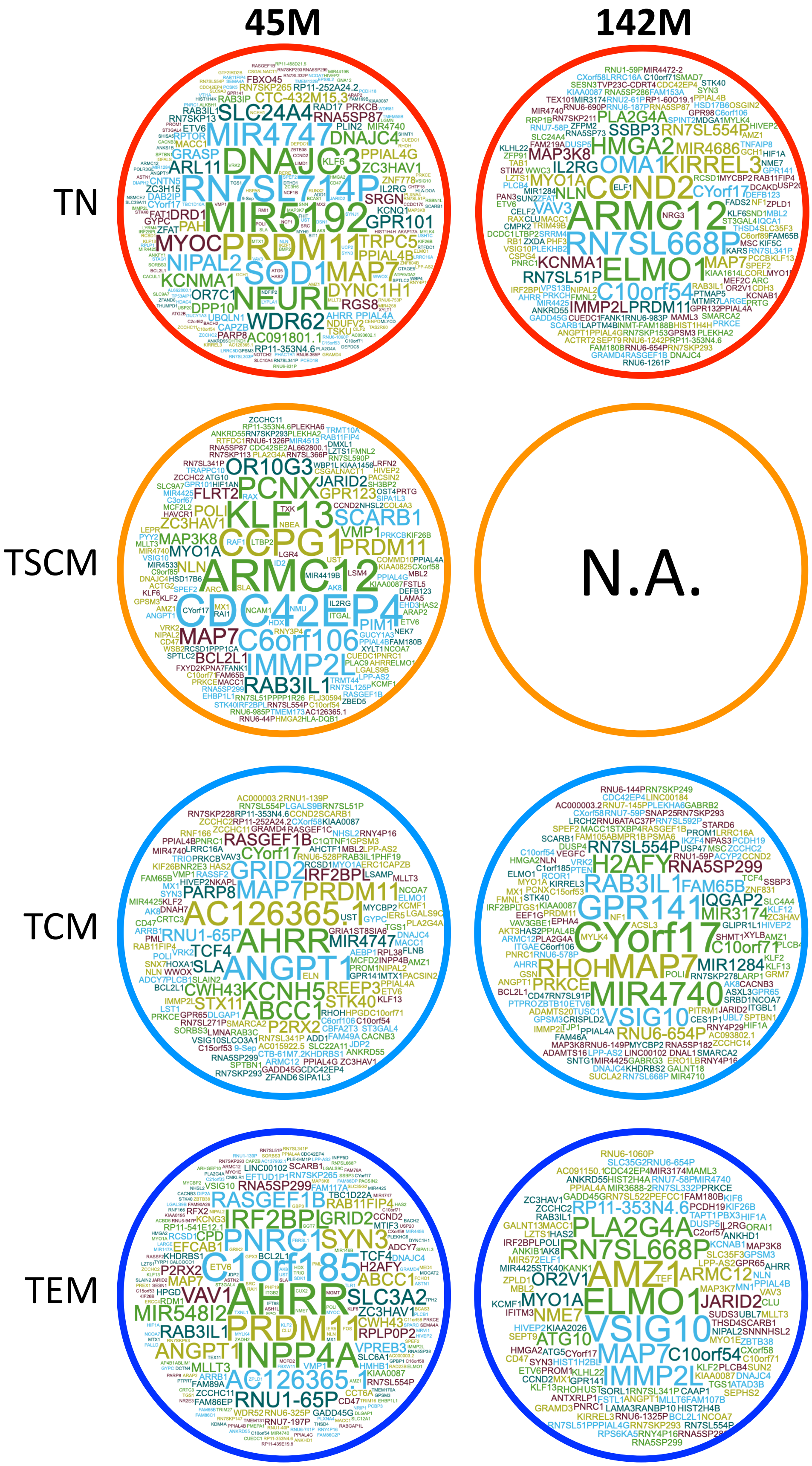

Supplementary Figure 12 Word clouds showing the genomic distribution of IS from P10 in each sample and timepoint. Relative size of each gene is proportional to the relative level of IS clustering (the bigger it is the gene name the higher it is the number of IS detected in its proximity).

AIIP

TN

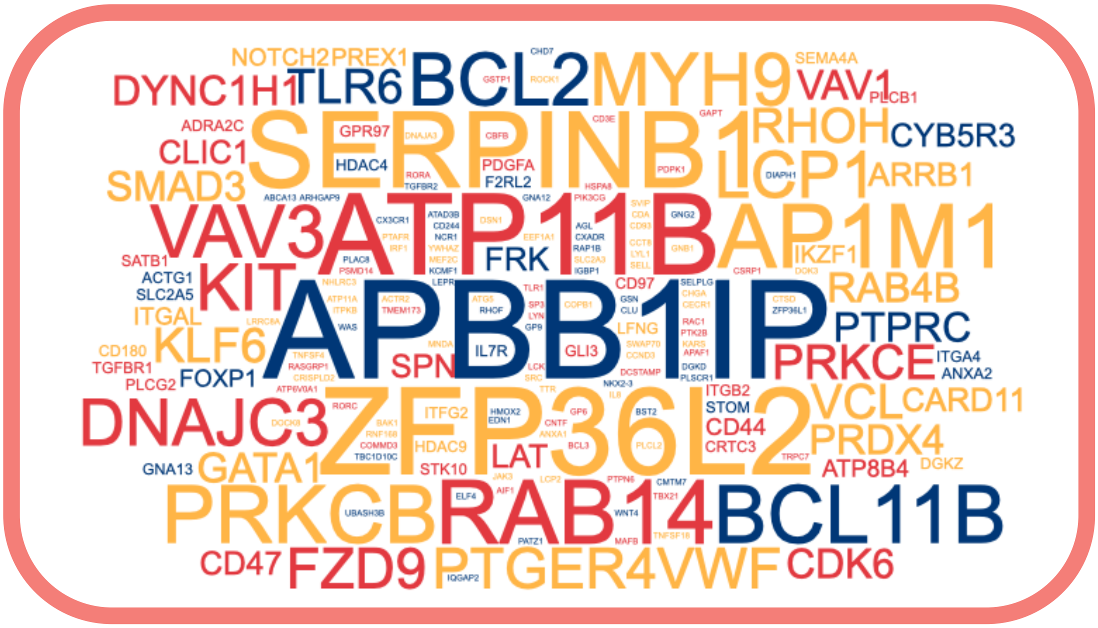

TN

Top10 GO Biological processes

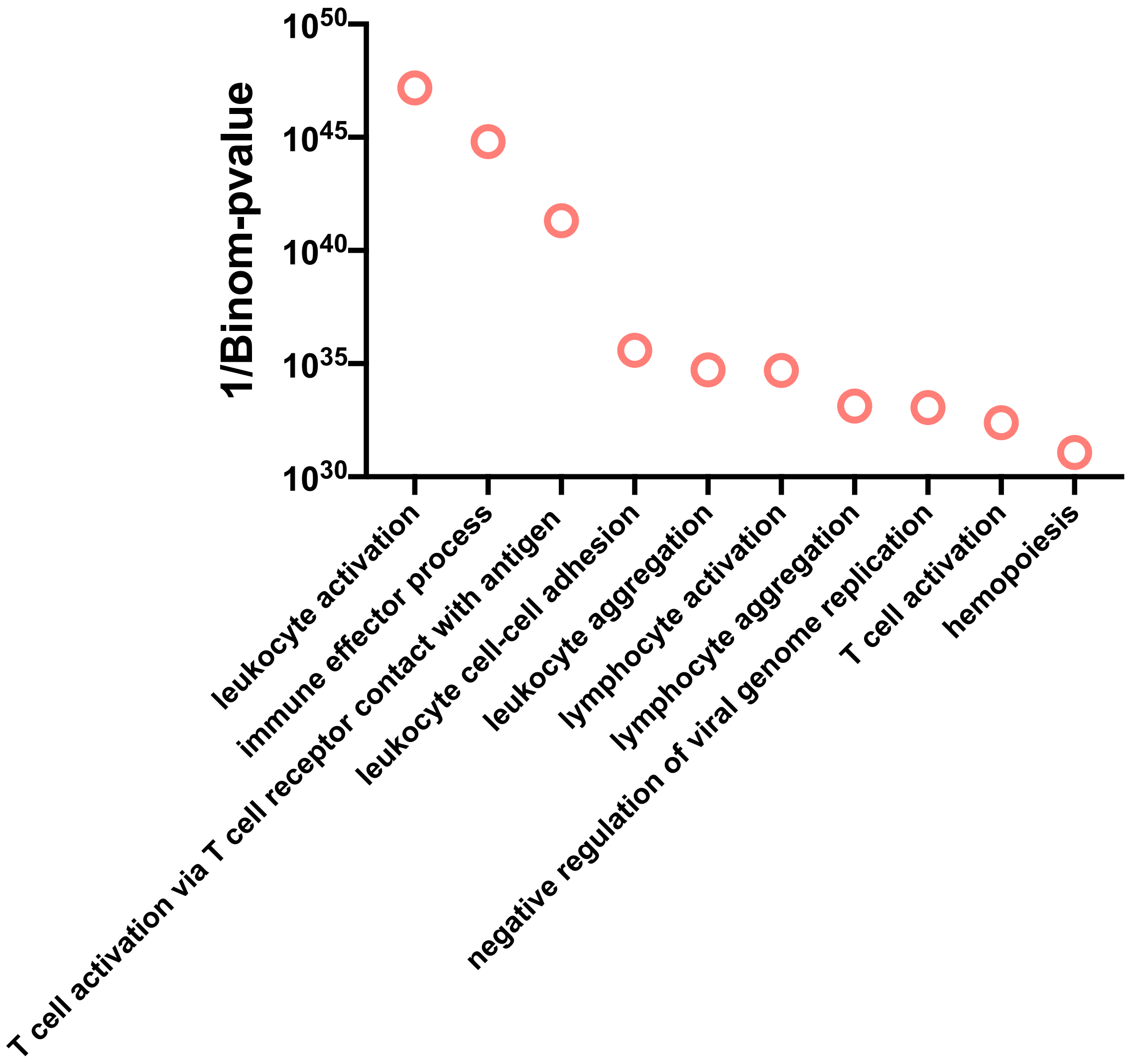

Supplementary Figure 13 Distribution of IS in TN and gene ontology of hit genes. The word cloud on the top show the genomic distribution of IS in TN of all patients and timepoint (graphical representation similar to Supplementary Fig. 6-10). The plot on the bottom show significance of the top Gene Ontology categories involved by IS through as analyzed through the Genomic Regions Enrichment of Annotation Tool (GREAT, great.stanford.edu).

FigS14

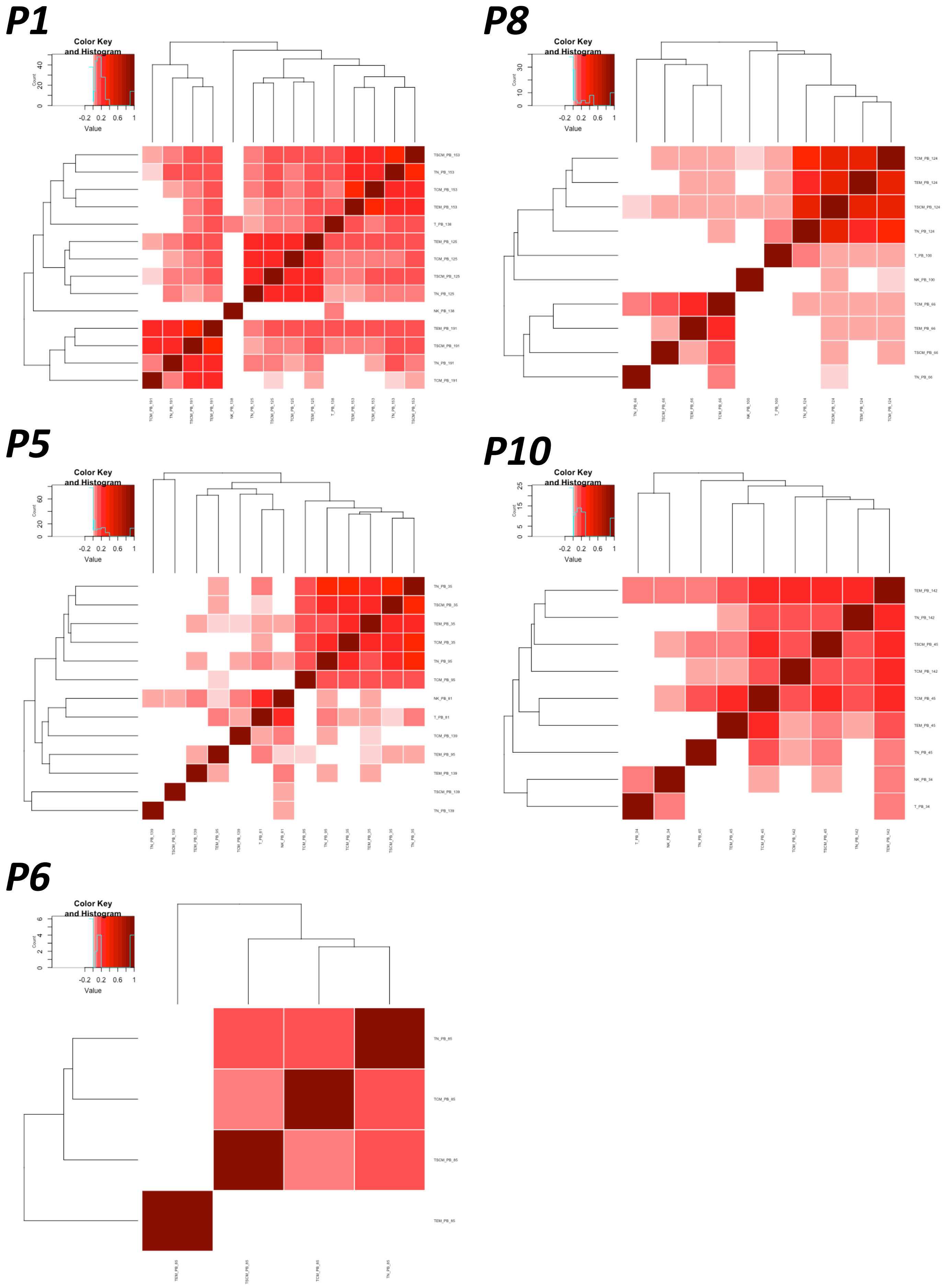

Supplementary Figure 14 Heatmaps showing Pearson Correlations Coefficients (scale of reds) across populations and timepoints in the 5 patients analyzed (column labels are shown as "subpopulation_PB(peripheral blood)_months after GT").

FigS15

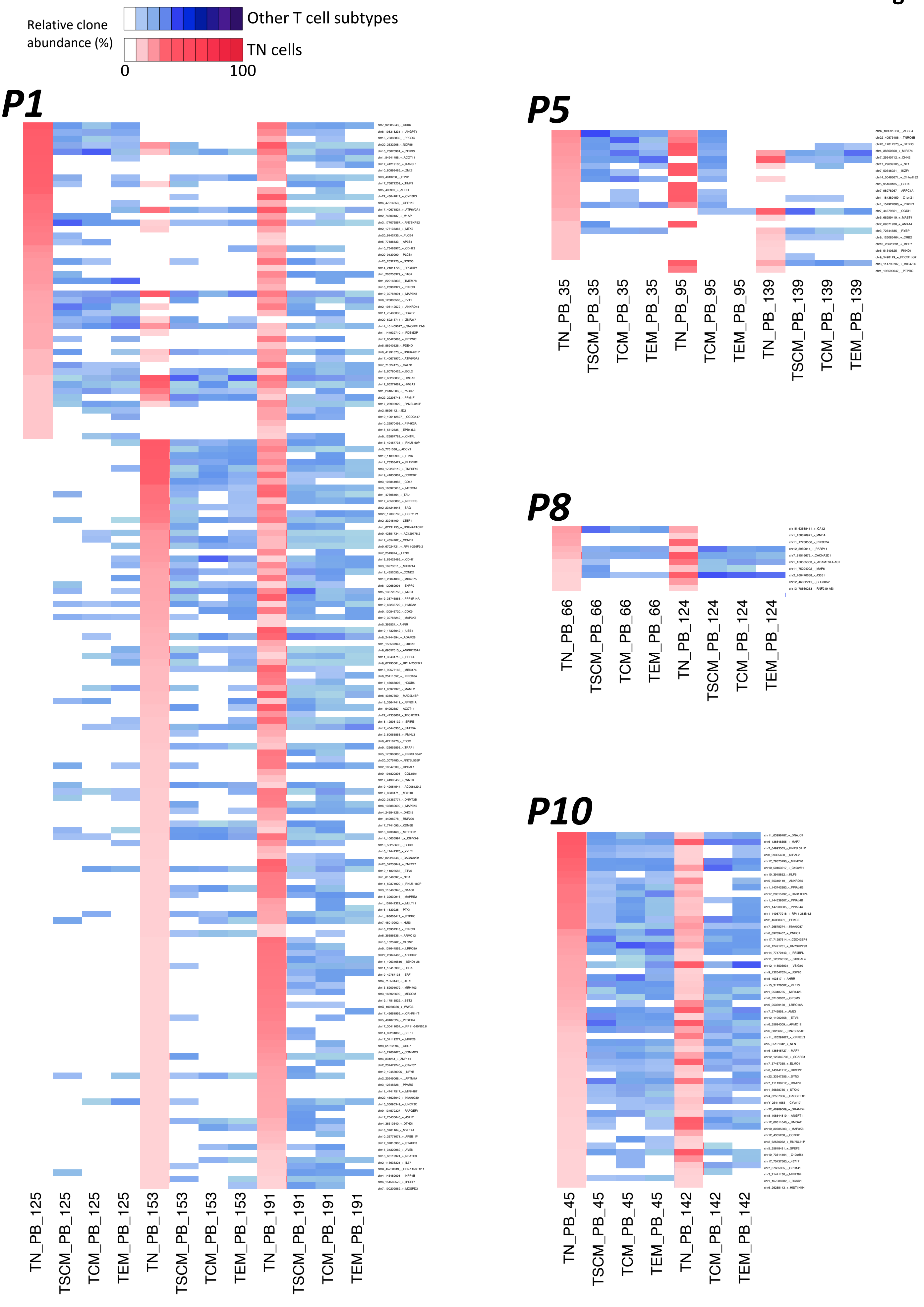

Relative clone abundance (%)
Other T cell subtypes
TN cells
0
100
P1
TN_PB_125
TSCM_PB_125
TCM_PB_125
TEM_PB_125
TN_PB_153
TSCM_PB_153
TCM_PB_153
TEM_PB_153
TN_PB_191
TSCM_PB_191
TCM_PB_191
TEM_PB_191
P5
TN_PB_35
TSCM_PB_35
TCM_PB_35
TEM_PB_35
TN_PB_95
TCM_PB_95
TEM_PB_95
TN_PB_139
TSCM_PB_139
TCM_PB_139
TEM_PB_139
P8
TN_PB_66
TSCM_PB_66
TCM_PB_66
TEM_PB_66
TN_PB_124
TSCM_PB_124
TCM_PB_124
TEM_PB_124
P10
TN_PB_45
TSCM_PB_45
TCM_PB_45
TEM_PB_45
TN_PB_142
TCM_PB_142
TEM_PB_142

Supplementary Figure 15 Heatmaps displaying recaptured TN integration sites in each patient across each sample/timepoint. Each row is named by the locus of the IS and the name of the closest gene while each column is labeled by sample and timepoint (number of months after GT). Intensity of color is proportional to the abundance of each integration site (red = IS from TN, blue = IS from other T cell subtypes, white = IS not detected).

FigS16

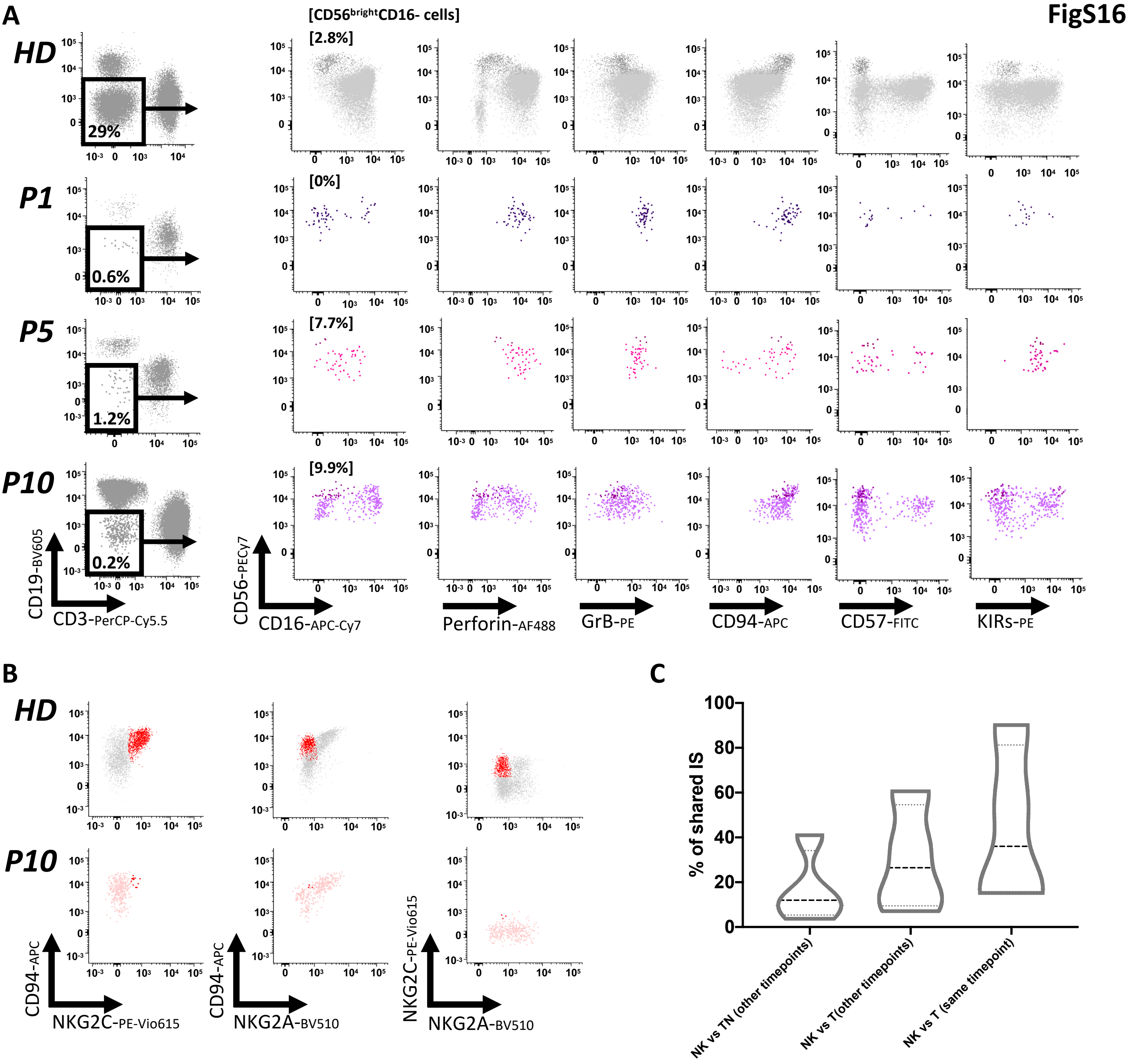
A
[CD56brightCD16- cells]
HD
29%
[2.8%]
P1
0.6%
[0%]
P5
1.2%
[7.7%]
P10
0.2%
[9.9%]
CD19-BV605
CD3-PerCP-Cy5.5
CD56-PECy7
CD16-APC-Cy7
Perforin-AF488
GrB-PE
CD94-APC
CD57-FITC
KIRs-PE
B
HD
P10
CD94-APC
NKG2C-PE-Vio615
CD94-APC
NKG2A-BV510
NKG2C-PE-Vio615
NKG2A-BV510
C
% of shared IS
100
80
60
40
20
0
NK vs TN (other timepoints)
NK vs T(other timepoints)
NK vs T (same timepoint)

Supplementary Figure 16 Immunophenotyping of NK cells. a) FACS plots showing the percentages of NK cells as well as the expression of CD56, CD16, Perforin, Granzyme B (GrB), CD94, CD57 and Killer-cells immunoglobulin-like receptors (KIRs) in NK cells in one HD as well as P1, P5 and P10. The percentage of $CD56^{bright}$CD16- within the NK population is shown for each individual and events falling into this gate are highlighted with a darker colour shade in each dot plot. b) FACS plots showing the identification of memory/adaptive NK cells (CD94+NKG2C+NKG2A-) in a healthy donor and in P10. e) Violin plots showing the percentage of identical IS shared between NK cells and TN at the different timepoints, NK cells and total T cells at different timepoints and NK cells and total T cells at the same timepoint.

Supplementary Table1

PT treated/ **PT analysed**	Year treated
**PT1**	**2001**
PT2	2001
PT3	2002
PT4	2002
**PT5**	**2003**
**PT6**	**2004**
PT7	2004
**PT8**	**2005**
PT9	2006
**PT10**	**2006**

Supplementary Table 1 List of patients treated and a date of their infusion. Patients that were part of this study are underlined in bold.

Supplementary Table2

Analysis of T cell subpopulations

Population	N. of cells used as input (range)
TN	48,528 – 1,000,000
TSCM	9,446 - 31,949
TCM	13,456 – 183,370
TEM	52,697 – 421,209

Analysis of total T cells and NK cells

Population	N. of cells used as input (range)
T cells	358,500 – 1,200,000
NK cells	20,000 – 30,650

Supplementary Table 2 Top table shows ranges of the amount of sorted T cell populations that were used as an input for IS analysis. Bottom table shows ranges of the amount of sorted NK and total T cells that were used as an input for IS analysis.

Supplementary Table3

**P1**	TN_PB_125	TSCM_PB_125	TCM_PB_125	TEM_PB_125	NK_PB_138	T_PB_138	TN_PB_153	TSCM_PB_153	TCM_PB_153	TEM_PB_153	TN_PB_191	TSCM_PB_191	TCM_PB_191	TEM_PB_191	Total unique
Unique IS	111	134	153	176	272	398	256	250	159	370	932	869	1671	308	3489
SeqReads	*30788*	*103627*	*136919*	*121099*	*2993541*	*5395665*	*184905*	*118143*	*59056*	*225758*	*201103*	*244016*	*290563*	*3464*	

**P5**	TN_PB_35	TSCM_PB_35	TCM_PB_35	TEM_PB_35	NK_PB_81	T_PB_81	TN_PB_95	TCM_PB_95	TEM_PB_95	TN_PB_139	TSCM_PB_139	TCM_PB_139	TEM_PB_139	Total unique
Unique IS	60	54	64	64	61	733	19	177	41	1612	1437	1119	567	4832
SeqReads	*24797*	*32978*	*85908*	*82332*	*77610*	*949293*	*1215*	*74411*	*76062*	*279892*	*351830*	*311558*	*440569*	

**P6**	TN_PB_85	TSCM_PB_85	TCM_PB_85	TEM_PB_85	Total unique
Unique IS	144	151	177	615	889
SeqReads	*443196*	*182873*	*315687*	*139665*	

**P8**	TN_PB_66	TSCM_PB_66	TCM_PB_66	TEM_PB_66	NK_PB_100	T_PB_100	TN_PB_124	TSCM_PB_124	TCM_PB_124	TEM_PB_124	Total unique
Unique IS	475	376	193	376	296	723	50	23	43	45	2053
SeqReads	*196184*	*288614*	*205853*	*363884*	*3136071*	*2408961*	*67609*	*43496*	*28552*	*11484*	

**P10**	NK_PB_34	T_PB_34	TN_PB_45	TSCM_PB_45	TCM_PB_45	TEM_PB_45	TN_PB_142	TCM_PB_142	TEM_PB_142	Total unique
Unique IS	22	348	391	225	213	399	221	206	172	1493
SeqReads	*174501*	*2084619*	*217882*	*242955*	*89282*	*299170*	*238597*	*91780*	*149742*	

Supplementary Table 3 Number of IS collected from each patient and timepoint and relative total reads count (Total unique = total IS from each patient after removing redundancies of shared IS across samples).

Supplementary Table4

P1	Shannon	Simpson	inverseSimpson
TN_PB_125	1.954513	0.758108	4.13407
TSCM_PB_125	1.958153	0.748482	3.97586
TCM_PB_125	2.018748	0.73249	3.73818
TEM_PB_125	2.380668	0.826052	5.74886
NK_PB_138	0.928342	0.489361	1.95833
T_PB_138	2.118468	0.834229	6.03242
TN_PB_153	2.28076	0.856156	6.95197
TSCM_PB_153	2.382301	0.803025	5.07678
TCM_PB_153	1.179195	0.602138	2.51343
TEM_PB_153	2.303175	0.763762	4.23302
TN_PB_191	3.459834	0.888968	9.00644
TSCM_PB_191	3.075799	0.841886	6.32454
TCM_PB_191	4.952752	0.971851	35.52527
TEM_PB_191	4.506504	0.975202	40.32645
**P5**	**Shannon**	**Simpson**	**inverseSimpson**
TN_PB_35	0.167195	0.043499	1.04548
TSCM_PB_35	0.285738	0.092753	1.10224
TCM_PB_35	1.532516	0.722655	3.60562
TEM_PB_35	1.44854	0.649688	2.8546
NK_PB_81	0.094356	0.021445	1.02191
T_PB_81	1.437778	0.420116	1.72448
TN_PB_95	2.033158	0.828058	5.81592
TCM_PB_95	1.151242	0.611504	2.57403
TEM_PB_95	1.759823	0.776474	4.47375
TN_PB_139	3.039742	0.923808	13.12472
TSCM_PB_139	2.061177	0.802362	5.05975
TCM_PB_139	2.113851	0.827814	5.80767
TEM_PB_139	1.9478	0.808906	5.23303
**P6**	**Shannon**	**Simpson**	**inverseSimpson**
TN_PB_85	1.57646	0.705355	3.39392
TSCM_PB_85	1.35031	0.647462	2.83657
TCM_PB_85	1.11664	0.575549	2.35598
TEM_PB_85	1.44596	0.659548	2.93727
**P8**	**Shannon**	**Simpson**	**inverseSimpson**
TN_PB_66	2.176592	0.80094	5.02361
TSCM_PB_66	2.404198	0.85375	6.8376
TCM_PB_66	1.10513	0.493098	1.97277
TEM_PB_66	2.19712	0.837083	6.13809
NK_PB_100	0.991327	0.556119	2.25286
T_PB_100	0.960098	0.35621	1.5533
TN_PB_124	1.704508	0.723694	3.61918
TSCM_PB_124	0.909627	0.453047	1.82831
TCM_PB_124	1.941166	0.780557	4.55699
TEM_PB_124	1.660852	0.754719	4.07696
**P10**	**Shannon**	**Simpson**	**inverseSimpson**
NK_PB_34	0.710519	0.455293	1.83585
T_PB_34	1.397988	0.696314	3.29288
TN_PB_45	1.969127	0.771187	4.37038
TSCM_PB_45	2.022389	0.826966	5.7792
TCM_PB_45	1.658898	0.655994	2.90693
TEM_PB_45	2.615692	0.882173	8.48699
TN_PB_142	1.766004	0.756545	4.10753
TCM_PB_142	2.232625	0.826362	5.75911
TEM_PB_142	1.564321	0.636525	2.75122

Supplementary Table 4 Diversity of each subpopulation and timepoint (number of months after GT) calculated by means of Shannon, Simpson and InverseSimpson diversity indexes.

Supplementary Table5

P1 IS recaptures within TN

Number of captured units: **1118**

Abundance estimations and model fits:

	abundance	stderr	deviance	df	AIC	BIC
M0	2955.8	177.9	1083.509	5	1127.708	1137.747
Mt	1972.6	98.4	22.641	3	70.84	90.917
Mh Chao (LB)	3149.8	216.9	1078.348	4	1124.547	1139.605
Mh Poisson2	4034.5	648	1078.348	4	1124.547	1139.605
Mh Darroch	5304.4	1524.4	1078.348	4	1124.547	1139.605
Mh Gamma3.5	7155.6	3096.3	1078.348	4	1124.547	1139.605
**Mth Chao (LB)**	**2092.6**	**118.8**	**11.292**	**2**	**61.491**	**86.588**
Mth Poisson2	2823.5	391.6	11.292	2	61.491	86.588
Mth Darroch	4102.7	1105.8	11.292	2	61.491	86.588
Mth Gamma3.5	6379.4	2736.2	11.292	2	61.491	86.588
Mb	-53	10.7	63.774	4	109.973	125.031
Mbh	4.3	22.1	57.521	3	105.72	125.797

P5 IS recaptures within TN

Number of captured units: **1667**

Abundance estimations and model fits:

	abundance	stderr	deviance	df	AIC	BIC
M0	39526.2	7915.2	3173.327	5	3209.065	3219.903
Mt	5339.3	905.1	79.719	3	119.457	141.132
Mh Chao (LB)	46767.4	10329.3	3165.027	4	3202.765	3219.021
Mh Poisson2	225698.5	128462.9	3165.027	4	3202.765	3219.021
Mh Darroch	1114519.8	1087794.1	3165.027	4	3202.765	3219.021
Mh Gamma3.5	5646209.5	7877070.8	3165.027	4	3202.765	3219.021
**Mth Chao (LB)**	**6056.6**	**1155.2**	**69.11**	**2**	**110.848**	**137.942**
Mth Poisson2	31449.3	17633.6	69.11	2	110.848	137.942
Mth Darroch	203730.7	201410.9	69.11	2	110.848	137.942
Mth Gamma3.5	1407210.4	1994449.9	69.11	2	110.848	137.942
Mb	-0.6	0.8	304.095	4	341.833	358.09
Mbh	60	0.2	0.204	3	39.942	61.617

Supplementary Table 5 Number of captured IS units in TN of P1 and P5 overtime and abundance estimations based on IS re-capturing using different models (in bold the results reported in Fig. 5E).

Supplementary Table6

Marker	Fluorochrome	Clone	Source	Cat number	Volume (uL/sample)	Tube
**CD16**	APC-Cy7	3G8	Biolegend	302017	5	1&2
**CD56**	PECy7	5.1H11	Biolegend	362509	5	1&2
**CD45**	BV650	HI30	BD Horizon	563717	2.5	1&2
**CD3**	PerCP-Cy5.5	UCHT1	BD Pharmingen	560835	5	1&2
**CD19**	BV605	HIB19	Biolegend	302243	5	1&2
**CCR7**	BV510	3D12	BD Horizon	563449	5	1
**CCR5**	BV711	J418F1	Biolegend	359129	5	1
**CD158a***	PE	HP-MA4	Biolegend	339505	5	1
**CD158b***	PE	DX27	Biolegend	312605	5	1
**CD158e1***	PE	DX9	Biolegend	312707	5	1
**CD158i***	PE	REA860	Miltenyi Biotec	130-114-772	2	1
**CX3CR1**	PE/Dazzle 594	2A9-1	Biolegend	341623	5	1
**CCR2**	BV421	K036C2	Biolegend	357209	5	1
**CXCR3**	AF647	G025H7	Biolegend	353711	5	1
**CD57**	FITC	HNK-1	BD	333169	20	1
**CD27**	BV421	M-T271	BD Horizon	562514	2	2
**CD127**	BV711	HIL-7R-M21	BD Horizon	563165	5	2
**Perforin**	AF488	δG9	BD Pharmingen	563764	5	2
**Granzyme B**	PE	GB11	BD Pharmingen	561142	5	2
**CD94**	APC	HP-3D9	BD Pharmingen	559876	20	2
**NKG2C****	PE-Vio615	REA205	Miltenyi Biotec	130-123-047	2	2#
**NKG2A****	BV510	131411	BD OptiBuild	747922	5	2#
**CD132#**	PE	TUGh4	Biolegend	338605	5	3

*All KIRs were measured together in the same fluorochrome.
** These antibodies were added to the tube 2 to analyse the memory/adaptive NK cells in patient 10 and two healthy donors.
# used for IL2RG Common gamma chain expression in T and NK cells

Supplementary Table 6 NK cell immunophenotyping antibody panel.

Supplementary Table7

Marker	Fluorochrome	Clone	Source	Cat number	Volume* (uL/sample)
**CD8**	Pe-Cy5	RPA-T8	BD Pharmigen	555368	2
**CD45RA**	V450	HI100	BD Horizon	560362	2
**CD62L**	APC	DREG-56	Biolegend	304810	3
**CD3**	V500	UCHT1	BD Horizon	561416	5
**CD95**	PE	DX2	Biolegend	305608	4
**CD4**	APC-Cy7	RPA-T4	BD Pharmigen	55787	2
**CD3/CD16+CD56+ /CD45/CD19**	FITC/PE/PerCP/APC	SJ25C1/SK7/B73.1/NCAM16.2/2D1	BD Multitest	342416	30
**CD14**	APC-Cy7	MφP9	BD Biosciences	557831	7
**IFN-gamma**	FITC	B27	BD Pharmigen	557718	5**
**CD3/CD16+CD56+ /CD45/CD19/CD4/ CD8**	FITC/PE/PerCP-Cy5.5/APC/PE-Cy7/APC-Cy7	SK7/B73.1/NCAM16.2/2D1/SJ25C1/SK3/SK1	BD Multitest	644611	30

*Diluted in a total volume of 100 uL PBS
**Diluted in a total volume of 50 uL of BD Perm/Wash buffer

Supplementary Table 7 T cell subsets immunophenotyping and IFN-gamma production assay antibody panels.

Supplementary Table 8

Method	Sequence	Name
**qPCR/ddPCR**	5' TGC TAA AAC TGC AGA ATC TGG T 3'	Gamma Chain (gc) Forward Primer
**qPCR/ddPCR**	5' AGC TGG GAT TCAC TCA GTT TG 3'	Gamma Chain (gc) Reverse Primer
**ddPCR**	5' **FAM**- CCT GGG CTC AGA GAA CCT AAC A -**ZEN** 3'	Gamma chain (gc) Probe
**qPCR**	5' **FAM**- CCT GGG CTC AGA GAA CCT AAC A -**TAMRA** 3'	Gamma chain (gc) Probe
**qPCR/ddPCR**	5’ TGA AGG TGG AGG ACA TTC CTC TA 3’	ApoB Forward Primer
**qPCR/ddPCR**	5’ CTG GAA TTG CGA TTT CTG GTA A 3’	ApoB Reverse Primer
**ddPCR**	5’ **HEX**- CGA GAA TCA CCC TGC CAG ACT TCC GT -**ZEN** 3’	ApoB Probe
**qPCR**	5’ **VIC**- CGA GAA TCA CCC TGC CAG ACT TCC GT - **TAMRA** 3’	ApoB Probe
**LAM-PCR**	(5’BIOTIN) AGCTGTTCCATCTGTTCCTGACCTT3'	Linear amplification step **-** 5'MLV I BIOT
**LAM-PCR**	(5’BIOTIN) TGCTTACCACAGATATCCTG 3’	Linear amplification step **-** 5'MLV II BIOT
**LAM-PCR**	5’ GACCTTGATCTGAACTTCTC 3’	1° Exponential PCR - retroLTR III
**LAM-PCR**	5’ GACCCGGGAGATCTGAATTC 3’	1° Exponential PCR - LCP I
**LAM-PCR**	5’ TTCCATGCCTTGCAAAATGGC 3’	2° Exponential PCR - retroLTR IV
**LAM-PCR**	5’ GATCTGAATTCAGTGGCACAG 3’	2° Exponential PCR - LCP II
**LAM-PCR**	5' GACCCGGGAGATCTGAATTCAGTG GCACAGCAGTTAGGNNNNNNGTAA GGNNNNNNAGATCTGGAATGAACT GGCC 3'	Linker cassette - LC1
**LAM-PCR**	5' AATTGGCCAGTTCATTCCAGATCT 3'	Linker cassette – LC3
**LAM-PCR**	5' CGGGCCAGTTCATTCCAGATCT 3'	Linker cassette – LC5
**TREC**	5’ CAC ATC CCT TTC AAC CAT GCT 3’	Forward primer
**TREC**	5’ TGC AGG TGC CTA TGC ATC A 3’	Reverse primer
**TREC analysis RNAseP**	5’ AGATTTGGACCTGCGAGCG 3’	Forward primer
**TREC analysis RNAseP**	5’ GAGCGGCTGTCTCCACAAGT 3’	Reverse primer

Supplementary Table 8 List of primers used for qPCR/digital PCR (ddPCR) for VCN evaluation, LAM-PCR and TREC analyses.

Supplementary Table 9

LAM-PCR. Illumina Fusion Primers for FUSION PRIMER PCR

LTR Specific Primers

Name	Sequence	Barcode
GR-P5-Rd1-LTR.1	AATGATACGGCGACCACCGAGATCTACACTCTTTCCCTACACGACGCTCTTCCGATCTNNNNNNNNNNNNAAACATCGCCTTGCAAAATGGCGTTACT	AAACATCG
GR-P5-Rd1-LTR.2	AATGATACGGCGACCACCGAGATCTACACTCTTTCCCTACACGACGCTCTTCCGATCTNNNNNNNNNNNNAACAACCACCTTGCAAAATGGCGTTACT	AACAACCA
GR-P5-Rd1-LTR.3	AATGATACGGCGACCACCGAGATCTACACTCTTTCCCTACACGACGCTCTTCCGATCTNNNNNNNNNNNNAACCGAGACCTTGCAAAATGGCGTTACT	AACCGAGA
GR-P5-Rd1-LTR.4	AATGATACGGCGACCACCGAGATCTACACTCTTTCCCTACACGACGCTCTTCCGATCTNNNNNNNNNNNNAACGCTTACCTTGCAAAATGGCGTTACT	AACGCTTA
GR-P5-Rd1-LTR.5	AATGATACGGCGACCACCGAGATCTACACTCTTTCCCTACACGACGCTCTTCCGATCTNNNNNNNNNNNNAACGTGATCCTTGCAAAATGGCGTTACT	AACGTGAT
GR-P5-Rd1-LTR.6	AATGATACGGCGACCACCGAGATCTACACTCTTTCCCTACACGACGCTCTTCCGATCTNNNNNNNNNNNNAACTCACCCCTTGCAAAATGGCGTTACT	AACTCACC
GR-P5-Rd1-LTR.7	AATGATACGGCGACCACCGAGATCTACACTCTTTCCCTACACGACGCTCTTCCGATCTNNNNNNNNNNNNAAGACGGACCTTGCAAAATGGCGTTACT	AAGACGGA
GR-P5-Rd1-LTR.8	AATGATACGGCGACCACCGAGATCTACACTCTTTCCCTACACGACGCTCTTCCGATCTNNNNNNNNNNNNAAGAGATCCCTTGCAAAATGGCGTTACT	AAGAGATC
GR-P5-Rd1-LTR.9	AATGATACGGCGACCACCGAGATCTACACTCTTTCCCTACACGACGCTCTTCCGATCTNNNNNNNNNNNNAAGGACACCCTTGCAAAATGGCGTTACT	AAGGACAC
GR-P5-Rd1-LTR.10	AATGATACGGCGACCACCGAGATCTACACTCTTTCCCTACACGACGCTCTTCCGATCTNNNNNNNNNNNNAAGGTACACCTTGCAAAATGGCGTTACT	AAGGTACA

Supplementary Table 9 LAM-PCR. Illumina Fusion Primers for FUSION PRIMER PCR. LTR Specific Primers

Supplementary Table 10

LAM-PCR. Illumina Fusion Primers for FUSION PRIMER PCR

LC Specific Primers

Name	Sequence	Barcode
GR-P7-Rd2.LC.1	CAAGCAGAAGACGGCATACGAGATGTGACTGGAGTTCAGACGTGTGCTCTTCCGATCTNNNNNNNNNNNNCAACCACAgatctgaattcagtggcacag	CAACCACA
GR-P7-Rd2.LC.2	CAAGCAGAAGACGGCATACGAGATGTGACTGGAGTTCAGACGTGTGCTCTTCCGATCTNNNNNNNNNNNNCAAGACTAgatctgaattcagtggcacag	CAAGACTA
GR-P7-Rd2.LC.3	CAAGCAGAAGACGGCATACGAGATGTGACTGGAGTTCAGACGTGTGCTCTTCCGATCTNNNNNNNNNNNNCAAGGAGCgatctgaattcagtggcacag	CAAGGAGC
GR-P7-Rd2.LC.4	CAAGCAGAAGACGGCATACGAGATGTGACTGGAGTTCAGACGTGTGCTCTTCCGATCTNNNNNNNNNNNNCAATGGAAgatctgaattcagtggcacag	CAATGGAA
GR-P7-Rd2.LC.5	CAAGCAGAAGACGGCATACGAGATGTGACTGGAGTTCAGACGTGTGCTCTTCCGATCTNNNNNNNNNNNNCACCTTACgatctgaattcagtggcacag	CACCTTAC
GR-P7-Rd2.LC.6	CAAGCAGAAGACGGCATACGAGATGTGACTGGAGTTCAGACGTGTGCTCTTCCGATCTNNNNNNNNNNNNCACTTCGAgatctgaattcagtggcacag	CACTTCGA
GR-P7-Rd2.LC.7	CAAGCAGAAGACGGCATACGAGATGTGACTGGAGTTCAGACGTGTGCTCTTCCGATCTNNNNNNNNNNNNCAGATCTGgatctgaattcagtggcacag	CAGATCTG
GR-P7-Rd2.LC.8	CAAGCAGAAGACGGCATACGAGATGTGACTGGAGTTCAGACGTGTGCTCTTCCGATCTNNNNNNNNNNNNCAGCGTTAgatctgaattcagtggcacag	CAGCGTTA
GR-P7-Rd2.LC.9	CAAGCAGAAGACGGCATACGAGATGTGACTGGAGTTCAGACGTGTGCTCTTCCGATCTNNNNNNNNNNNNCATACCAAgatctgaattcagtggcacag	CATACCAA
GR-P7-Rd2.LC.10	CAAGCAGAAGACGGCATACGAGATGTGACTGGAGTTCAGACGTGTGCTCTTCCGATCTNNNNNNNNNNNNCATCAAGTgatctgaattcagtggcacag	CATCAAGT

Supplementary Table 10 LAM-PCR. Illumina Fusion Primers for FUSION PRIMER PCR. LC Specific Primers

Supplementary Table 11

Vbeta spectratyping

Name	Sequence
Cβ primer	GGGTGTGGGAGATCTCTGC
Cβ primer (FAM labelled)	ACACAGCAGCCTCGGGTGGG
Vβ1	CCGCACAACAGTTCCCTGACTTGC
Vβ2	CACAACTATGTTTTGGTATCGTC
Vβ3	CGCTTCTCCCTGATTCTGGAGTCC
Vβ4	TTCCCATCAGCCGCCCAAACCTAA
Vβ5	GATCAAAACGAGAGGACAGC
Vβ6a	GATCCAATTTCAGGTCATACTG
Vβ6b1	CAGGGSCCAGAGTTTCTGAC
Vβ6b2	AGGGCTCAGAGGTTCTGAC
Vβ7	CCTGAATGCCCCAACAGCTCT
Vβ8	GGTACAGACAGACCATGATGC
Vβ9	TTCCCTGGAGCTTGGTGACTCTGC
Vβ11	GTCAACAGTCTCCAGAATAAGG
Vβ12	TCCYCCTCACTCTGGAGTC
Vβ13a	GGTATCGACAAGACCCAGGCA
Vβ13b	AGGCTCATCCATTATTCAAATAC
Vβ14	GGGCTGGGCTTAAGGCAGATCTAC
Vβ15	CAGGCACAGGCTAAATTCTCCCTG
Vβ16	GCCTGCAGAACTGGAGGATTCTGG
Vβ17	TCCTCTCACTGTGACATCGGCCCA
Vβ18	CTGCTGAATTTCCCAAAGAGGGCC
Vβ20	TGCCCAGAATCTCTCAGCCTCCA
Vβ21	GGAGTAGACTCCACTCTCAAG
Vβ22	GATCCGGTCCACAAAGCTGG
Vβ23	ATTCTGAACTGAACATGAGCTCCT
Vβ24	GACATCCGCTCACCAGGCCTG

Supplementary Table 11 Oligos used for Vbeta spectratyping

Supplementary Table 12

TCR Sequencing. Stage 1 primer sequences

TCR gamma

Name	Sequence
VG1-FR2	GTGACTGGAGTTCAGACGTGTGCTCTTCCGATCTGGAAGGCCCCACAGCRTCTT
VG9-FR2	GTGACTGGAGTTCAGACGTGTGCTCTTCCGATCTCGGCACTGTCAGAAAGGAATC
VG10-FR2	GTGACTGGAGTTCAGACGTGTGCTCTTCCGATCTAGCATGGGTAAGACAAGCAA
VG11-FR2	GTGACTGGAGTTCAGACGTGTGCTCTTCCGATCTCTTCCACTTCCACTTTGAAA
JG1.1-FR2	ACACTCTTTCCCTACACGACGCTCTTCCGATCTTTACCAGGCGAAGTTACTATGAGC
JG1.3-FR2	ACACTCTTTCCCTACACGACGCTCTTCCGATCTGTGTTGTTCCACTGCCAAAGAG

TCR delta

Name	Sequence
VD1-FR2	GTGACTGGAGTTCAGACGTGTGCTCTTCCGATCTATGCAAAAAGTGGTCGCTATT
VD2-FR2	GTGACTGGAGTTCAGACGTGTGCTCTTCCGATCTATACCGAGAAAAGGACATCTATG
VD3-FR2	GTGACTGGAGTTCAGACGTGTGCTCTTCCGATCTGTACCGGATAAGGCCAGATTA
VD4-FR2	GTGACTGGAGTTCAGACGTGTGCTCTTCCGATCTATGACCAGCAAAATGCAACAG
VD5-FR2	GTGACTGGAGTTCAGACGTGTGCTCTTCCGATCTACCCTGCTGAAGGTCCTACAT
VD6-FR2	GTGACTGGAGTTCAGACGTGTGCTCTTCCGATCTCCCTGCATTATTGATAGCCAT
DD2F-FR2	GTGACTGGAGTTCAGACGTGTGCTCTTCCGATCTAGCGGGTGGTGATGGCAAAGT
JD1-FR2	ACACTCTTTCCCTACACGACGCTCTTCCGATCTGTTCCACAGTCACACGGGTTC
JD2-FR2	ACACTCTTTCCCTACACGACGCTCTTCCGATCTGTTCCACGATGAGTTGTGTTC
JD3-FR2	ACACTCTTTCCCTACACGACGCTCTTCCGATCTCTCACGGGGCTCCACGAAGAG
JD4-FR2	ACACTCTTTCCCTACACGACGCTCTTCCGATCTTTGACCTCCAGATAGGTTCC
DD3R-FR2	ACACTCTTTCCCTACACGACGCTCTTCCGATCTTGGGACCCAGGGTGAGGATAT
Ja29-FR2	ACACTCTTTCCCTACACGACGCTCTTCCGATCTGGCAAAAGCATTCTAGGTACA
Ja9-FR2	ACACTCTTTCCCTACACGACGCTCTTCCGATCTTTTAACTGGCAGACAAAACTATG
Ja30-FR2	ACACTCTTTCCCTACACGACGCTCTTCCGATCTGCCACCCACATGTCTTAG
Ja48-FR2	ACACTCTTTCCCTACACGACGCTCTTCCGATCTTCCCCAGAATCTTATGCAG
Ja54-FR2	ACACTCTTTCCCTACACGACGCTCTTCCGATCTGAGGGGCAAGTAATTAAATCA
Ja58-FR2	ACACTCTTTCCCTACACGACGCTCTTCCGATCTGACTTGAATGTGGCAGAGA
Ja61-FR2	ACACTCTTTCCCTACACGACGCTCTTCCGATCTGTTTGTTAAGGCACATTAGAATC

Supplementary Table 12 TCR Sequencing. Stage 1 primer sequences. TCR gamma and TCR delta

Supplementary Table 13

TCR Sequencing. Stage 1 primer sequences

TCR beta

Name	Sequence
Vβ2-FR2	GTGACTGGAGTTCAGACGTGTGCTCTTCCGATCTAACTATGTTTTGGTATCGTCA
Vβ4-FR2	GTGACTGGAGTTCAGACGTGTGCTCTTCCGATCTCACGATGTTCTGGTACCGTCAGCA
Vβ5/1-FR2	GTGACTGGAGTTCAGACGTGTGCTCTTCCGATCTCAGTGTGTCCTGGTACCAACAG
Vβ6a/11-FR2	GTGACTGGAGTTCAGACGTGTGCTCTTCCGATCTAACCCTTTATTGGTACCGACA
Vβ6b/25-FR2	GTGACTGGAGTTCAGACGTGTGCTCTTCCGATCTATCCCTTTTTTGGTACCAACAG
Vβ6c-FR2	GTGACTGGAGTTCAGACGTGTGCTCTTCCGATCTAACCCTTTATTGGTATCAACAG
Vβ7-FR2	GTGACTGGAGTTCAGACGTGTGCTCTTCCGATCTCGCTATGTATTGGTACAAGCA
Vβ8a-FR2	GTGACTGGAGTTCAGACGTGTGCTCTTCCGATCTCTCCCGTTTTCTGGTACAGACAGAC
Vβ9-FR2	GTGACTGGAGTTCAGACGTGTGCTCTTCCGATCTCGCTATGTATTGGTATAAACAG
Vβ10-FR2	GTGACTGGAGTTCAGACGTGTGCTCTTCCGATCTTTATGTTTACTGGTATCGTAAGAAGC
Vβ11-FR2	GTGACTGGAGTTCAGACGTGTGCTCTTCCGATCTCAAAATGTACTGGTATCAACAA
Vβ12a/3a/13a/15-FR2	GTGACTGGAGTTCAGACGTGTGCTCTTCCGATCTATACATGTACTGGTATCGACAAGAC
Vβ13b-FR2	GTGACTGGAGTTCAGACGTGTGCTCTTCCGATCTGGCCATGTACTGGTATAGACAAG
Vβ13c/12b/14-FR2	GTGACTGGAGTTCAGACGTGTGCTCTTCCGATCTGTATATGTCCTGGTATCGACAAGA
Vβ16-FR2	GTGACTGGAGTTCAGACGTGTGCTCTTCCGATCTTAACCTTTATTGGTATCGACGTGT
Vβ17-FR2	GTGACTGGAGTTCAGACGTGTGCTCTTCCGATCTGGCCATGTACTGGTACCGACA
Vβ18-FR2	GTGACTGGAGTTCAGACGTGTGCTCTTCCGATCTTCATGTTTACTGGTATCGGCAG
Vβ19-FR2	GTGACTGGAGTTCAGACGTGTGCTCTTCCGATCTTTATGTTTATTGGTATCAACAGAATCA
Vβ20-FR2	GTGACTGGAGTTCAGACGTGTGCTCTTCCGATCTCAACCTATACTGGTACCGACA
Vβ21-FR2	GTGACTGGAGTTCAGACGTGTGCTCTTCCGATCTTACCCTTTACTGGTACCGGCAG
Vβ22-FR2	GTGACTGGAGTTCAGACGTGTGCTCTTCCGATCTATACTTCTATTGGTACAGACAAATCT
Vβ23/8b-FR2	GTGACTGGAGTTCAGACGTGTGCTCTTCCGATCTCACGGTCTACTGGTACCAGCA
Vβ24-FR2	GTGACTGGAGTTCAGACGTGTGCTCTTCCGATCTCGTCATGTACTGGTACCAGCA
Dβ1-FR2	GTGACTGGAGTTCAGACGTGTGCTCTTCCGATCTGCCAAACAGCCTTACAAAGAC
Dβ2-FR2	GTGACTGGAGTTCAGACGTGTGCTCTTCCGATCTTTTCCAAGCCCCACACAGTC
Jβ1.1-FR2	ACACTCTTTCCCTACACGACGCTCTTCCGATCTCTTACCTACAACTGTGAATCTGGTG
Jβ1.2-FR2	ACACTCTTTCCCTACACGACGCTCTTCCGATCTCTTACCTACAACGGTTAACCTGGTC
Jβ1.3-FR2	ACACTCTTTCCCTACACGACGCTCTTCCGATCTCTTACCTACAACAGTGAGCCAACTT
Jβ1.4-FR2	ACACTCTTTCCCTACACGACGCTCTTCCGATCTCATACCCAAGACAGAGAGCTGGGTTC
Jβ1.5-FR2	ACACTCTTTCCCTACACGACGCTCTTCCGATCTCTTACCTAGGATGGAGAGAGTCGAGTC
Jβ1.6-FR2	ACACTCTTTCCCTACACGACGCTCTTCCGATCTCATACCTGTCACAGTGAGCCTG
Jβ2.1-FR2	ACACTCTTTCCCTACACGACGCTCTTCCGATCTCCTTCTTACCTAGCACGGTGA
Jβ2.2-FR2	ACACTCTTTCCCTACACGACGCTCTTCCGATCTCTTACCCAGTACGGTCAGCCT
Jβ2.3-FR2	ACACTCTTTCCCTACACGACGCTCTTCCGATCTCCCGCTTACCGAGCACTGTCA
Jβ2.4-FR2	ACACTCTTTCCCTACACGACGCTCTTCCGATCTCCAGCTTACCCAGCACTGAGA
Jβ2.5-FR2	ACACTCTTTCCCTACACGACGCTCTTCCGATCTCGCGCACACCGAGCAC
Jβ2.6-FR2	ACACTCTTTCCCTACACGACGCTCTTCCGATCTCTCGCCCAGCACGGTCAGCCT
Jβ2.7-FR2	ACACTCTTTCCCTACACGACGCTCTTCCGATCTCTTACCTGTAACCGTGAGCCTG

Supplementary Table 13 TCR Sequencing. Stage 1 primer sequences. TCR beta

Supplementary Table 14

TCR Sequencing. Stage 2 primer sequences

Name	Sequence	Index in oligonucleotide	Index red sequence
i7_01	CAAGCAGAAGACGGCATACGAGATTCTAGCTAGTGACTGGAGTTCAGACGTGTGCTCTTCCGATC*T	TCTAGCTA	TAGCTAGA
i7_02	CAAGCAGAAGACGGCATACGAGATCTAGCTATGTGACTGGAGTTCAGACGTGTGCTCTTCCGATC*T	CTAGCTAT	ATAGCTAG
i7_03	CAAGCAGAAGACGGCATACGAGATAGGTTGGCGTGACTGGAGTTCAGACGTGTGCTCTTCCGATC	AGGTTGGC	GCCAACCT
i7_04	CAAGCAGAAGACGGCATACGAGATGACCAACGGTGACTGGAGTTCAGACGTGTGCTCTTCCGATC*T	GACCAACG	CGTTGGTC
i7_05	CAAGCAGAAGACGGCATACGAGATGCGGAGTTGTGACTGGAGTTCAGACGTGTGCTCTTCCGATC*T	GCGGAGTT	AACTCCGC
i7_06	CAAGCAGAAGACGGCATACGAGATGTGCCATAGTGACTGGAGTTCAGACGTGTGCTCTTCCGATC*T	GTGCCATA	TATGGCAC
i7_07	CAAGCAGAAGACGGCATACGAGATTAATGTCCGTGACTGGAGTTCAGACGTGTGCTCTTCCGATC*T	TAATGTCC	GGACATTA
i7_08	CAAGCAGAAGACGGCATACGAGATCGAAGGACGTGACTGGAGTTCAGACGTGTGCTCTTCCGATC*T	CGAAGGAC	GTCCTTCG
i7_09	CAAGCAGAAGACGGCATACGAGATAATGTCCTGTGACTGGAGTTCAGACGTGTGCTCTTCCGATC*T	AATGTCCT	AGGACATT
i7_10	CAAGCAGAAGACGGCATACGAGATAGAACATTGTGACTGGAGTTCAGACGTGTGCTCTTCCGATC*T	AGAACATT	AATGTTCT
i7_11	CAAGCAGAAGACGGCATACGAGATTGTCAGTCGTGACTGGAGTTCAGACGTGTGCTCTTCCGATC*T	TGTCAGTC	GACTGACA
i7_12	CAAGCAGAAGACGGCATACGAGATCACCGCTTGTGACTGGAGTTCAGACGTGTGCTCTTCCGATC*T	CACCGCTT	AAGCGGTG
i7_13	CAAGCAGAAGACGGCATACGAGATCAGACGCAGTGACTGGAGTTCAGACGTGTGCTCTTCCGATC*T	CAGACGCA	TGCGTCTG
i7_14	CAAGCAGAAGACGGCATACGAGATGCTACTAGGTGACTGGAGTTCAGACGTGTGCTCTTCCGATC*T	GCTACTAG	CTAGTAGC
i7_15	CAAGCAGAAGACGGCATACGAGATGTCAGTCTGTGACTGGAGTTCAGACGTGTGCTCTTCCGATC*T	GTCAGTCT	AGACTGAC
i7_16	CAAGCAGAAGACGGCATACGAGATTTCACCGCGTGACTGGAGTTCAGACGTGTGCTCTTCCGATC*T	TTCACCGC	GCGGTGAA
i7_17	CAAGCAGAAGACGGCATACGAGATGGTCTAATGTGACTGGAGTTCAGACGTGTGCTCTTCCGATC*T	GGTCTAAT	ATTAGACC
i7_18	CAAGCAGAAGACGGCATACGAGATACCTGGATGTGACTGGAGTTCAGACGTGTGCTCTTCCGATC*T	ACCTGGAT	ATCCAGGT
i7_19	CAAGCAGAAGACGGCATACGAGATAGCGACAGGTGACTGGAGTTCAGACGTGTGCTCTTCCGATC*T	AGCGACAG	CTGTCGCT
i7_20	CAAGCAGAAGACGGCATACGAGATATAGGCTCGTGACTGGAGTTCAGACGTGTGCTCTTCCGATC*T	ATAGGCTC	GAGCCTAT
i7_21	CAAGCAGAAGACGGCATACGAGATTAGAACATGTGACTGGAGTTCAGACGTGTGCTCTTCCGATC*T	TAGAACAT	ATGTTCTA
i7_22	CAAGCAGAAGACGGCATACGAGATTGCGGAGTGTGACTGGAGTTCAGACGTGTGCTCTTCCGATC*T	TGCGGAGT	ACTCCGCA
i7_23	CAAGCAGAAGACGGCATACGAGATTTGCGGAGGTGACTGGAGTTCAGACGTGTGCTCTTCCGATC*T	TTGCGGAG	CTCCGCAA
i7_24	CAAGCAGAAGACGGCATACGAGATTTAGAACAGTGACTGGAGTTCAGACGTGTGCTCTTCCGATC*T	TTAGAACA	TGTTCTAA
i5_01	AATGATACGGCGACCACCGAGATCTACACCACTTGAGACACTCTTTCCCTACACGACGCTCTTCCGATC*T	CACTTGAG	CACTTGAG
i5_02	AATGATACGGCGACCACCGAGATCTACACGTTACCGAACACTCTTTCCCTACACGACGCTCTTCCGATC*T	GTTACCGA	GTTACCGA
i5_03	AATGATACGGCGACCACCGAGATCTACACTGACGACTACACTCTTTCCCTACACGACGCTCTTCCGATC*T	TGACGACT	TGACGACT
i5_04	AATGATACGGCGACCACCGAGATCTACACACGGATTCACACTCTTTCCCTACACGACGCTCTTCCGATC*T	ACGGATTC	ACGGATTC
i5_05	AATGATACGGCGACCACCGAGATCTACACCCATAGGAACACTCTTTCCCTACACGACGCTCTTCCGATC*T	CCATAGGA	CCATAGGA
i5_06	AATGATACGGCGACCACCGAGATCTACACTGGAAGGCACACTCTTTCCCTACACGACGCTCTTCCGATC*T	TGGAAGGC	TGGAAGGC
i5_07	AATGATACGGCGACCACCGAGATCTACACGCATCATGACACTCTTTCCCTACACGACGCTCTTCCGATC*T	GCATCATG	GCATCATG
i5_08	AATGATACGGCGACCACCGAGATCTACACAGCGGTGAACACTCTTTCCCTACACGACGCTCTTCCGATC*T	AGCGGTGA	AGCGGTGA
i5_09	AATGATACGGCGACCACCGAGATCTACACAGTTACCGACACTCTTTCCCTACACGACGCTCTTCCGATC*T	AGTTACCG	AGTTACCG
i5_10	AATGATACGGCGACCACCGAGATCTACACCATGCATAACACTCTTTCCCTACACGACGCTCTTCCGATC*T	CATGCATA	CATGCATA
i5_11	AATGATACGGCGACCACCGAGATCTACACACATGCATACACTCTTTCCCTACACGACGCTCTTCCGATC*T	ACATGCAT	ACATGCAT
i5_12	AATGATACGGCGACCACCGAGATCTACACACCATAGGACACTCTTTCCCTACACGACGCTCTTCCGATC*T	ACCATAGG	ACCATAGG
i5_13	AATGATACGGCGACCACCGAGATCTACACTCCAGGTAACACTCTTTCCCTACACGACGCTCTTCCGATC*T	TCCAGGTA	TCCAGGTA
i5_14	AATGATACGGCGACCACCGAGATCTACACCTTAATTGACACTCTTTCCCTACACGACGCTCTTCCGATC*T	CTTAATTG	CTTAATTG
i5_15	AATGATACGGCGACCACCGAGATCTACACCGGATTCAACACTCTTTCCCTACACGACGCTCTTCCGATC*T	CGGATTCA	CGGATTCA
i5_16	AATGATACGGCGACCACCGAGATCTACACTTAGACCAACACTCTTTCCCTACACGACGCTCTTCCGATC*T	TTAGACCA	TTAGACCA

Supplementary Table 14 TCR Sequencing. Stage 2 primer sequences.

Supplementary Table 15

TCR Sequencing. Stage 2 indexing oligos – Group1

Index Grouping One			
i7	Index read sequence	i5	Index read sequence
i7_01	TAGCTAGA	i5_01	CACTTGAG
i7_03	GCCAACCT	i5_02	GTTACCGA
i7_04	CGTTGGTC	i5_03	TGACGACT
i7_05	AACTCCGC	i5_04	ACGGATTC
i7_06	TATGGCAC	i5_05	CCATAGGA
i7_07	GGACATTA	i5_06	TGGAAGGC
i7_08	GTCCTTCG	i5_07	GCATCATG
i7_10	AATGTTCT	i5_08	AGCGGTGA
i7_11	GACTGACA	i5_10	CATGCATA
i7_12	AAGCGGTG	i5_13	TCCAGGTA
i7_13	TGCGTCTG	i5_14	CTTAATTG
i7_14	CTAGTAGC	i5_16	TTAGACCA
i7_17	ATTAGACC		
i7_18	ATCCAGGT		
i7_19	CTGTCGCT		
i7_20	GAGCCTAT		

Supplementary Table 15 TCR Sequencing. Stage 2 indexing oligos. Group 1

Supplementary Table 16

TCR Sequencing. Stage 2 indexing oligos – Group2

Index Grouping Two			
i7	Index read sequence	i5	Index read sequence
i7_02	ATAGCTAG	i5_01	CACTTGAG
i7_03	GCCAACCT	i5_03	TGACGACT
i7_04	CGTTGGTC	i5_06	TGGAAGGC
i7_06	TATGGCAC	i5_07	GCATCATG
i7_08	GTCCTTCG	i5_08	AGCGGTGA
i7_09	AGGACATT	i5_09	AGTTACCG
i7_13	TGCGTCTG	i5_11	ACATGCAT
i7_14	CTAGTAGC	i5_12	ACCATAGG
i7_15	AGACTGAC	i5_13	TCCAGGTA
i7_16	GCGGTGAA	i5_14	CTTAATTG
i7_17	ATTAGACC	i5_15	CGGATTCA
i7_18	ATCCAGGT	i5_16	TTAGACCA
i7_19	CTGTCGCT		
i7_20	GAGCCTAT		
i7_21	ATGTTCTA		
i7_22	ACTCCGCA		

Supplementary Table 16 TCR Sequencing. Stage 2 indexing oligos. Group 2
