# Supplementary material for: Long-term lymphoid progenitors independently sustain naïve T and NK cell production in humans
Source: Nat Commun. 2021 Mar 12;12:1622. doi: 10.1038/s41467-021-21834-9 (PMC7954865; doi:10.1038/s41467-021-21834-9)
Supplement: Supplementary file 2 — Reporting Summary [file 41467_2021_21834_MOESM2_ESM.pdf]

nature research

Corresponding author(s): Luca Biasco

Last updated by author(s): 23/01/2021

Reporting Summary

Nature Research wishes to improve the reproducibility of the work that we publish. This form provides structure for consistency and transparency in reporting. For further information on Nature Research policies, see our Editorial Policies and the Editorial Policy Checklist.

Statistics

For all statistical analyses, confirm that the following items are present in the figure legend, table legend, main text, or Methods section.

n/a	Confirmed	
☐	☒	The exact sample size (*n*) for each experimental group/condition, given as a discrete number and unit of measurement
☐	☒	A statement on whether measurements were taken from distinct samples or whether the same sample was measured repeatedly
☐	☒	The statistical test(s) used AND whether they are one- or two-sided<br>*Only common tests should be described solely by name; describe more complex techniques in the Methods section.*
☒	☐	A description of all covariates tested
☒	☐	A description of any assumptions or corrections, such as tests of normality and adjustment for multiple comparisons
☐	☒	A full description of the statistical parameters including central tendency (e.g. means) or other basic estimates (e.g. regression coefficient) AND variation (e.g. standard deviation) or associated estimates of uncertainty (e.g. confidence intervals)
☐	☒	For null hypothesis testing, the test statistic (e.g. *F*, *t*, *r*) with confidence intervals, effect sizes, degrees of freedom and *P* value noted<br>*Give P values as exact values whenever suitable.*
☒	☐	For Bayesian analysis, information on the choice of priors and Markov chain Monte Carlo settings
☒	☐	For hierarchical and complex designs, identification of the appropriate level for tests and full reporting of outcomes
☒	☐	Estimates of effect sizes (e.g. Cohen's *d*, Pearson's *r*), indicating how they were calculated

Our web collection on statistics for biologists contains articles on many of the points above.

Software and code

Policy information about availability of computer code

Data collection	Immunophenotyping: FACS raw data were collected through DIVA software (BD Bioscience v8.0.1).<br><br>IS analysis: Custom code required for IS mapping was already described in Biasco et al. Cell Stem Cell 2016. Raw integration sites data sets underwent series of different bioinformatics filtering procedures according to the type of analysis to be performed. All data sets were processed with a “collision detection filter” to univocally assign each IS to a patient and to one or more T cell subpopulations by applying a 10-fold rule for contamination identification as previously reported. A final matrix was generated where each row represented an individual integration site while each column an individual cell type/sample and time point.<br><br>Additional specifications are reported in the Methods section
Data analysis	Immunophenotyping: Raw data was acquired using FACSDiva software (v8.0.1) and analysed using either FlowJo (TreeStar v10) (T cell new panel, IFN-gamma assay), Summit software (no version available, analyses date back to 2001) (BD Biosciences) (T cells old panel) or Infinicyt v2.0 for the NK panel (Cytognos SL).<br>Graphical representations of VCN and lymphocyte counts were generated with Prism (v8, GraphPad Software). VCN analysis has been done either in Bio-Rad CFX Manager v3.1 (qPCR) or QuantaSoft v1.7.4.0917 (ddPCR).<br>We included reference to the SPA based software that was used for Vbeta spectratyping analysis. No official version is available for this software.

For manuscripts utilizing custom algorithms or software that are central to the research but not yet described in published literature, software must be made available to editors and reviewers. We strongly encourage code deposition in a community repository (e.g. GitHub). See the Nature Research guidelines for submitting code & software for further information.

Data

Policy information about availability of data

All manuscripts must include a data availability statement. This statement should provide the following information, where applicable:

- Accession codes, unique identifiers, or web links for publicly available datasets
- A list of figures that have associated raw data
- A description of any restrictions on data availability

Raw data containing TCR rearrangements and IS are provided as .csv supplementary material files

Field-specific reporting

Please select the one below that is the best fit for your research. If you are not sure, read the appropriate sections before making your selection.

[x] Life sciences [ ] Behavioural & social sciences [ ] Ecological, evolutionary & environmental sciences

For a reference copy of the document with all sections, see nature.com/documents/nr-reporting-summary-flat.pdf

Life sciences study design

All studies must disclose on these points even when the disclosure is negative.

Sample size	Sample size was constrained by limited clinical sample availability. For phenotypic characterization and IS analyses of T cell subsets in SCIDX1 patients we collected in vivo biological material available during years 2001-2018. We aimed at analyzing for all the patients at least 1 early and 1 late timepoint (where possible we did more) . For IS analysis of NK and T cell identical integrations we acquired only 1 timepoint per patient, for TCR sequencing we aimed to acquire 1 early and 1 late timepoints, for IFN-gamma assay we analysed samples from the latest timepoints available.
Data exclusions	All available healthy donors' and patients' samples are reported in the manuscript. Technically validated results were always included to the analyses and we did not apply any exclusion criteria for outliers.
Replication	For phenotypic characterization of T and NK cells, Rainbow beads (RB) calibration was performed during the set up of the instrumentation for FACS analyses. RB acquisition was performed before each sample acquisition in order to achieve reproducible instrument setting among different experiments. Both T and NK phenotype experiments were performed independently.<br>For VCN evaluation, qPCR was validated for reproducibility. We run each sample in triplicate and values are reported as mean of the three triplicates. All attempts at replication were successful.
Randomization	The experimental design did not include allocation of samples to randomised experimental group. We analyzed all the samples available during years 2001-2018
Blinding	The experimental design did not include allocation to groups nor to blinding. There was no expected results prior to perform these analyses, therefore blinding tests were not applicable.

Reporting for specific materials, systems and methods

We require information from authors about some types of materials, experimental systems and methods used in many studies. Here, indicate whether each material, system or method listed is relevant to your study. If you are not sure if a list item applies to your research, read the appropriate section before selecting a response.

Materials & experimental systems

n/a	Involved in the study	
[ ]	[x]	Antibodies
[x]	[ ]	Eukaryotic cell lines
[x]	[ ]	Palaeontology and archaeology
[x]	[ ]	Animals and other organisms
[ ]	[x]	Human research participants
[ ]	[x]	Clinical data
[x]	[ ]	Dual use research of concern

Methods

n/a	Involved in the study	
[x]	[ ]	ChIP-seq
[ ]	[x]	Flow cytometry
[x]	[ ]	MRI-based neuroimaging

Antibodies

Antibodies used	For each antibodies we described --> marker/fluorochrome/clone/manufacturer/cat.number<br>T cell subsets/IFN-gamma assay/sorting:

CD8 Pe-Cy5 RPA-T8 BD Pharmigen 555368
CD45RA V450 HI100 BD Horizon 560362
CD62L APC DREG-56 Biolegend 304810
CD3 V500 UCHT1 BD Horizon 561416
CD95 PE DX2 Biolegend 305608
CD4 APC-Cy7 RPA-T4 BD Pharmigen 55787
CD3/CD16+CD56+/CD45/CD19 FITC/PE/PerCP/APC SJ25C1/SK7/B73.1/NCAM16.2/2D1 BD Multitest 342416
CD3/CD16+CD56+/CD45/CD19/CD4/CD8 FITC/PE/PerCP-Cy5.5/APC/PE-Cy7/APC-Cy7 SK7/B73.1/NCAM16.2/2D1/SJ25C1/SK3/SK1 BD Multitest 644611

NK cells:
CD16 APC-Cy7 3G8 Biolegend 302017
CD56 PECy7 5.1H11 Biolegend 362509
CD45 BV650 HI30 BD Horizon 563717
CD3 PerCP-Cy5.5 UCHT1 BD Pharmingen 560835
CD19 BV605 HIB19 Biolegend 302243
CCR7 BV510 3D12 BD Horizon 563449
CCR5 BV711 J418F1 Biolegend 359129
CD158a* PE HP-MA4 Biolegend 339505
CD158b* PE DX27 Biolegend 312605
CD158e1* PE DX9 Biolegend 312707
CD158i* PE REA860 Miltenyi Biotec 130-114-772
CX3CR1 PE/Dazzle 594 2A9-1 Biolegend 341623
CCR2 BV421 K036C2 Biolegend 357209
CXCR3 AF647 G025H7 Biolegend 353711
CD57 FITC HNK-1 BD 333169
CD27 BV421 M-T271 BD Horizon 562514
CD127 BV711 HIL-7R-M21 BD Horizon 563165
Perforin AF488 δG9 BD Pharmingen 563764
Granzyme B PE GB11 BD Pharmingen 561142
CD94 APC HP-3D9 BD Pharmingen 559876

Validation

All the primary antibodies used for FACS staining are commercially available and have been validated for their use in human samples by the different manufacturers. For more specific information, please refer to manufacturers' websites using the aforementioned catalogue numbers.

Human research participants

Policy information about studies involving human research participants

Population characteristics

Five male SCIDX1 patients (average age 8 months at the time of infusion) for whom no human leukocyte antigen (HLA)-identical sibling donor or suitable matched unrelated donor was available, underwent gamma retroviral GT without any prior conditioning. All patients treated with RV GT in this study are alive and clinically most are well, including one patient who developed T-ALL at 24 months post GT, but after 2.5 years of
chemotherapy treatment went into clinical and molecular remission and remained there until the latest FU

Recruitment

Some patients with X-SCID were referred to GOSH through an existing network, in some cases patients with X-SCID at the research site and were already be known to the Investigators. Patients and their parents were approached by the Principal investigator and given a patient information leaflet, during this consultation the trial was discussed at length and patients have been given ample opportunity to ask questions. Once the patients are satisfied that they would like to enrol in to the trial, the principal Investigator would consent them and the process would be formally documented.

Ethics oversight

The Gene therapy advisory committee (GTAC in London) is UK national Research ethics committee dedicated for the review of gene therapy clinical research. GTAC had complete oversight of this trial

Note that full information on the approval of the study protocol must also be provided in the manuscript.

Clinical data

Policy information about clinical studies
All manuscripts should comply with the ICMJEguidelines for publication of clinical research and a completedCONSORT checklist must be included with all submissions.

Clinical trial registration

There was no requirement at the time to upload to public registries, so this is NA.

Study protocol	The protocol is located at the research site due to confidentiality reasons it is not a public accessible document
Data collection	The data was initially recorded in the source notes which would be paper based medical records. The laboratory data was recorded on a hospital based electronic system which was easily accessible for the research team.<br>The data was then transcribed by the study co-ordinator into paper based case report forms (CRF). Data was recorded in the CRF in an ongoing basis and then subsequently verified by the study monitor.
Outcomes	These were carefully defined during the design of the trial based on input from clinicians in this field. The outcomes were based on clinical importance to look at the effect of IMP. The outcome measures were looked at by analysing patient data periodically throughout the duration of the trial, and if required would have been amended. This manuscript does not pertain the clinical trial results therefore reporting of data regarding primary and secondary outcomes is not applicable in this context.

Flow Cytometry

Plots

Confirm that:

- [x] The axis labels state the marker and fluorochrome used (e.g. CD4-FITC).
- [x] The axis scales are clearly visible. Include numbers along axes only for bottom left plot of group (a 'group' is an analysis of identical markers).
- [x] All plots are contour plots with outliers or pseudocolor plots.
- [x] A numerical value for number of cells or percentage (with statistics) is provided.

Methodology

Sample preparation	Immunophenotyping was performed on whole blood EDTA samples and cell sorting was performed on PBMCs isolated from the whole blood by density gradient centrifugation using LymphoPrep (Sigma) after dextran sedimentation. Granulocytes were collected from the bottom of the lymphoprep tubes after centrifugation and red cell lysis was performed to remove red cell contaminants.<br>FACS sorting of T, B, NK cells and monocytes from PBMCs was performed on FACS Aria (BD Biosciences) analysed with FlowJo software (TreeStar).using the following antibody panel: CD3/CD56/CD16/CD19/CD45RA FITC/PE/APC/PerCP (Multitest; BD Biosciences) and CD14 APC-Cy7 (BD Biosciences). When feasible, an aliquot of the sorted cells was re-run through the cell sorter to check fraction purity.<br>Immunophenotyping of lymphocyte subsets was performed using a six colour multitest reagent (BD Biosciences) CD3 FITC, CD56/C16 PE, CD45 PerCP-Cy5.5, CD19 APC, CD4 PE-Cy7, and CD8-APC-Cy7. T cell immunophenotyping was performed using two antibody panels: a standard panel in place for routine clinical monitoring (CD45RA FITC, CD27 PE, CD45 PerCP, and CD4 or CD8 APC (BD Biosciences)) and a more comprehensive R&D panel CD3 V500 (BD Biosciences), CD95 PE (Biolegend), CD4 APC-Cy7 (BD Biosciences), CD8 PECy5 (BD Pharmigen), CD45RA V450 (BD Biosciences), CD62L APC (Biolegend)) developed to separate Naïve T cells, TSCMs, TCM, TEM and TEMRAs. This R&D panel was used to sort T cell subsets with FACS Aria or for immunophenotypic analysis of T cell subsets with Canto (after Rainbow bead calibration (Spherotech)). Raw FACS data was collected using DIVA software (BD Biosciences) and analysed with either Summit software for the clinical monitoring panel (BD Biosciences) or FlowJo for the R&D panel (TreeStar).<br>To study the NK cell phenotype, PBMCs were stained with the following conjugated antibodies in two different tubes (information about the clones and company is provided in supplementary Table 5): CCR2-BV421, CCR7-BV510, CD19-BV605, CD45-BV650, CCR5-BV711, CD57-FITC, CD3-PerCP-Cy5.5, KIRs -Killer cell immunoglobulin-like receptors- (CD158a, CD158b, CD158e and CD158i)-PE, CX3CR1 PE-Dazzle 594, CD56-PECy7, CXCR3-AF647, CD16 APC-Cy7, CD27-BV421, CD127-BV711, perforin-AF488, granzyme B-PE, CD94-APC. For the evaluation of cytoplasmic perforin and granzyme B, the Fix & Perm reagent kit (An der Grub, Vienna, Austria) was used, following manufacturer's instructions. All samples were acquired in a calibrated and compensated LSR II flow cytometer (BD Biosciences) and analysed with Infinicyt (Cytognos SL). NK cells were identified as lymphocytes CD45+, CD3-, CD19-, CD56 and/or CD16+. These NK cells were subclassified according the CD56 expression as CD56bright and CD56dim (Figure 5A).
Instrument	Sorting was done with FACS Aria. Immunophenotyping was done with BD FACSCanto (BD Biosciences) (after Rainbow bead calibration (Spherotech)).NK immunophenotyping was performed on calibrated and compensated LSR II flow cytometer (BD Biosciences).
Software	Raw FACS data was collected using DIVA software (BD Biosciences) and analysed with either FlowJo (TreeStar) and Infinicyt for the NK panel (Cytognos SL).
Cell population abundance	Where possible we measured purity of sorted cells and sorted cells reached a purity ranging between 92% and 99%. Due to the low amount of starting materials we run in parallel an<br>healthy donor sample to check the efficiency and purity of our sorting by acquiring sorted subpopulations at the analyzer.<br>Moreover, the double-sorting strategy allows an internal control of purity of our sorted populations.
Gating strategy	The gating strategy for new panel was shown in Figure 2. Briefly, after gating on singlets, we gated on Lymphocytes and then we used CD62L and CD45RA to identify DP precursors CD4/CD8+ CD3+CD45RA+CD62L+; Central memory (TCM) CD4/CD8+ CD3+CD45RA-CD62L+; and Effector memory (TEM) CD4/CD8+ CD3+CD45RA-CD62L- cells. We then used CD95 marker to identify Naïve T cells (TN) CD4/CD8+ CD3+CD45RA+CD62L+CD95- and T stem cell memory (TSCM) CD4/CD8+ CD3+CD45RA

+CD62L+CD95+ with the DP precursor population. NK cells were identified as lymphocytes CD45+, CD3-, CD19-, CD56 and/or CD16+. These NK cells were subclassified according the CD56 expression as CD56bright and CD56dim (Figure 5A).
For the old T cell panel: after gating on singlets, we gated on Lymphocytes and then we used CD62L and CD27 markers to identify DP precursors CD62L+CD27+, TCM CD62L+CD27- and TEM CD62L-CD27-.

☒ Tick this box to confirm that a figure exemplifying the gating strategy is provided in the Supplementary Information.
