# Supplementary material for: Long-term lymphoid progenitors independently sustain naïve T and NK cell production in humans
Source: Nat Commun. 2021 Mar 12;12:1622. doi: 10.1038/s41467-021-21834-9 (PMC7954865; doi:10.1038/s41467-021-21834-9)
Supplement: Supplementary file 3 — Description of Additional Supplementary Files [file 41467_2021_21834_MOESM3_ESM.pdf]

Description of Additional Supplementary Files

Supplementary Data 1 TCR dataset relative to Fig.3.

Supplementary Data 2 IS datasets relative to Fig.4-6.
